# Oxidative and reductive cyclization in stiff dithienylethenes

**DOI:** 10.3762/bjoc.14.259

**Published:** 2018-11-09

**Authors:** Michael Kleinwächter, Ellen Teichmann, Lutz Grubert, Martin Herder, Stefan Hecht

**Affiliations:** 1 Department of Chemistry & IRIS Adlershof, Humboldt-Universität zu Berlin, Brook-Taylor-Straße 2, 12489 Berlin, Germany

**Keywords:** diarylethenes, electrochromism, molecular switches, (spectro)electrochemistry

## Abstract

The electrochemical behavior of stiff dithienylethenes, undergoing double bond isomerization in addition to ring-closure, has been investigated. Electrochromism was observed in almost all cases, with the major pathway being the oxidatively induced cyclization of the open isomers. The influence of the ring size (to lock the reactive antiparallel conformation) as well as substituents (to modulate the redox potential) on the electrocyclization was examined. In the series of derivatives with 6-membered rings, both the *E-* and the *Z-*isomer convert to the closed isomer, whereas for the 7-membered rings no cyclization from the *E-*isomer was observed. For both stiff and normal dithienylethenes bearing benzonitrile substituents an additional and rare reductive electrocyclization was observed. The mechanism underlying both observed electrocyclization pathways has been elucidated.

## Introduction

Diarylethenes (DAEs) are a rich family of organic photoswitches formally derived from stilbene [1–2]. Upon irradiation they are able to undergo reversible photoisomerization based on 6π-electrocyclization and -cycloreversion, respectively, between two thermally stable states, which make them interesting components for optical memories [3–4]. In addition to photochemistry, the isomerization of DAEs can also be triggered by electrochemical means, therefore providing a stimulus orthogonal to light [5].

Electrochemically induced isomerization of DAEs is almost exclusively based on oxidation. Either cyclization [5–13] or cycloreversion [14–21] can be observed, while correlation of both reaction modes to the molecular structure is still under discussion [22–26]. There are only few reports about reductive isomerization, each involving the ionic methylpyridinium group as a substituent on the photochromic unit [27–29]. However, by a combination of suitable substituents, a bidirectional system able to operate in both switching directions either electrochemically or photochemically has been reported [28].

**Scheme 1:**
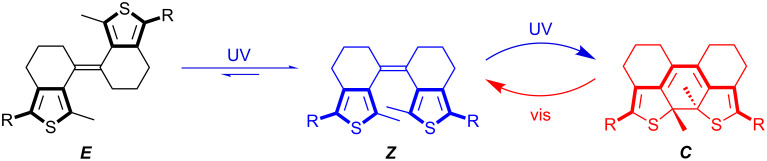
Combining double bond isomerization (***E***/***Z***) and cyclization/cycloreversion (***Z***/***C***) in three-state switching sDTEs. Photochemically, both ring-open isomers are converted to the closed isomer ***C*** in high yield when irradiated with near UV light. Upon irradiation with visible light the *C-*isomer undergoes quantitative cycloreversion exclusively to the *Z-*isomer [33].

We have recently developed a new subclass of DAEs without the geometric constraint of the central endocyclic olefin bridge yet with two rings each involving one of the bridge’s carbon atoms to lock the photoreactive antiparallel conformation and thus provide stiff dithienylethenes (sDTEs), in analogy to stiff stilbenes [30–32]. Due to enabled isomerization of the central exocyclic double bond, sDTEs form a three-state system undergoing interconversion between ring-open *E-* and *Z-*isomers and a ring-closed *C-*isomer (Scheme 1).

Here we describe the oxidatively and reductively induced isomerization behavior of sDTEs as investigated by cyclovoltammetry (CV) and spectro-electrochemistry (SEC) [33]. Our present study is aiming to: 1) elucidate the influence of possible double bond isomerization on the electrochromism of sDTEs; 2) explore the structural effect of varying ring size as well as electronic modification; and 3) contribute to the mechanistic understanding of electrochromism in DAEs in general.

**Scheme 2:**
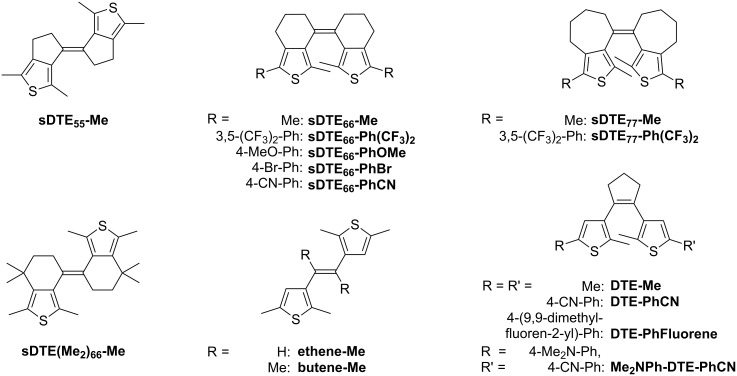
Overview of all sDTE and reference DTE compounds investigated in this study. The compound names indicate the molecular frame (e.g., sDTE_66_) and the substituents attached to the 5-position of the thiophenes (e.g., -PhOMe). For all compounds, the major isomer obtained in the synthesis [33] is shown.

First, we discuss the simple methyl-substituted sDTE derivative **sDTE_66_-Me**, consisting of two 6-membered rings, and subsequently relate to other members of this new family of compounds, possessing either different ring sizes or aromatic substituents with different electronic properties (Scheme 2). Interestingly, we found that oxidative cyclization can occur from both double bond isomers. In addition, a reductive cyclization was discovered in bis(benzonitrile)-substituted DTEs and also in a DTE with an extended π-system.

## Results and Discussion

### Cyclization by anodic oxidation

**Figure 1:**
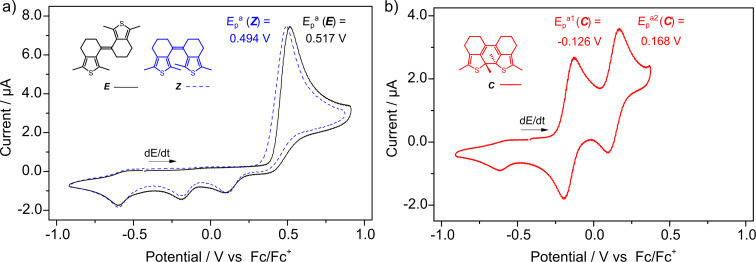
Cyclic voltammograms of **sDTE_66_-Me**. a) Both *E-* (black line) and *Z-*isomer (blue dashed line) display one irreversible oxidation wave, which is assigned to the formation of the closed isomer. b) The closed isomer (red line), generated in situ from ***Z-*sDTE_66_-Me** upon irradiation with 313 nm light prior to the CV measurement, displays two distinct oxidation waves. The first one is reversible whereas the second is quasi-reversible. Experiments were carried out in MeCN with 0.1 M Bu_4_NPF_6_, *c* = 1∙10^−3^ M, *dE*/*dt* = 1 V s^−1^.

In initial experiments, the electrochemical behavior of the methyl-substituted derivative bearing six-membered rings (**sDTE_66_-Me**) was investigated. For both configurational isomers, i.e., ***E-*** and ***Z-*sDTE_66_-Me**, an irreversible oxidation wave corresponding to the transfer of two electrons was observed in the cyclic voltammograms (Figure 1a), with the *Z-*isomer (blue dashed line) being slightly easier to oxidize than ***E-*sDTE_66_-Me** (black line). In contrast to both open isomers, two separate one-electron oxidation waves were observed for the closed isomer ***C-*sDTE_66_-Me** (Figure 1b), generated either in the irreversible oxidation process or photochemically. Both of these oxidation events occur at significantly lower potentials compared to the open isomers, reflecting the increased energy of the HOMO due to the extended π-system generated upon ring-closure.

Irreversible oxidation of the open isomer of DAEs has already been observed and was ascribed to cyclization [6–13]. Indeed, the two separate one-electron reduction waves arising during the back-sweep after oxidation of the *E-* and *Z-*isomer match exactly those of the independently photochemically prepared closed isomer (Figure 1b). Furthermore, in a second consecutive redox cycle (Figure S11, Supporting Information File 1) also two oxidation waves for ***C-*sDTE_66_-Me** are observed. Interestingly, oxidatively induced cyclization seems to occur similarly from both *Z-* and *E-*configured open isomers, i.e., regardless of the double bond geometry. Since ring-closure requires the two reactive α-thienyl carbon atoms to approach each other, presumably an additional electrochemically induced *E* → *Z* isomerization occurs prior to cyclization. Indeed, there are scattered reports about configurational isomerism in stilbene radical cations [34–35] and simple dithienylethenes [36–38]. As such, we postulate an equilibrium between both *E-* and *Z-*radical cations with the latter rapidly reacting to the closed isomer.

**Figure 2:**
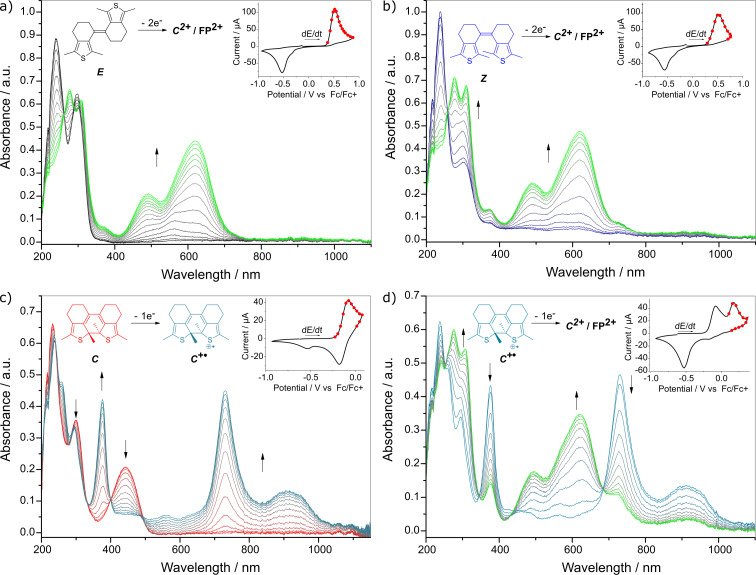
Spectroelectrochemistry of **sDTE_66_-Me**. Absorption changes during CV, insets showing the corresponding cyclovoltammograms with red dots marking when UV–vis–NIR spectra were measured. A stable intermediate is formed upon oxidation of: a) ***E-*sDTE_66_-Me** and b) ***Z-*sDTE_66_-Me**. c) Oxidation of photogenerated ***C-*sDTE_66_-Me** to the stable radical cation ***C^+•^***. d) Further oxidation of ***C^+•^*** to the dication **FP^2+^**, measured in a separate experiment. At the low sweep rates of SEC an irreversible reduction wave at *E*_p_^c^ = −0.530 V occurs in a), b), and d). Experiments were carried out in MeCN with 0.1 M Bu_4_NPF_6_, *c* = 5∙10^−4^ M, *dE*/*dt* = 10 mV s^−1^.

To gain a deeper mechanistic understanding of the oxidative cyclization, the evolution of the UV–vis absorption spectra during a CV cycle was recorded in a SEC cell. It was found that both ***E-*** and ***Z-*sDTE_66_-Me** convert from their colorless initial charge-neutral state into the same oxidized species which displays characteristic absorption bands centered at 493 nm and 621 nm (Figure 2a and b; for an overlay of spectra see Figure S29, Supporting Information File 1).

To identify the nature of this product, SEC was performed on the photochemically generated closed isomer (Figure 2c,d). Herein, the reversible first oxidation step yields the radical cation ***C^+•^*** (Figure 2c, light blue), identified by its characteristic red-shifted absorption at 731 nm and 912 nm due to an unpaired electron. The radical cation ***C^+•^*** is stable even at the slow scan rates of SEC and builds up continuously as evidenced by clean isosbestic points. In a subsequent, second oxidation step, the radical cation ***C^+•^*** is converted to the dication ***C*^2+^** (Figure 2d, green), as indicated by the hypsochromic shift due to the absence of an open-shell system. The formed dication exhibits characteristic absorption bands centered at 493 nm and 621 nm, identical to the species formed upon oxidation of the open isomers.

Notably, while the first oxidation step of the closed isomer (***C*** → ***C^+•^****)* is fully reversible, the second oxidation (***C^+•^*** → ***C*^2+^**) is only quasi-reversible. Formation of an unknown follow-up product (**FP**) occurs at the low scan rates of the SEC experiment, indicated by the appearance of a reduction wave at a significantly lower potential (*E*_p_^c^ = −0.530 V) during the return scan (Figure 2d). At the same time the reduction waves corresponding to the closed isomer, as observed in the CV experiment with high scan rates (Figure 1), disappear. Furthermore, the UV–vis spectrum recorded in the SEC after reduction is an overlay of that of the closed isomer and the new species **FP** possessing an absorbance maximum at 358 nm (Figure S29, Supporting Information File 1). This kind of irreversible process from an oxidized closed isomer has already been observed for DAEs [7], but its nature has not been further discussed except that it is different from the typical photochemical byproduct of DAEs [39–40].

Although the exact identity of **FP/FP^2+^** remains elusive, several characteristics can be summarized: 1) **FP^2+^** is formed from both the open and the closed isomers upon oxidation (Figure S41 and Figure S42, Supporting Information File 1) and its reduction wave is shifted to lower potentials compared to ***C^2+^*** for all observed cases by up to >600 mV (see Table S1, Supporting Information File 1). 2) The amount of **FP^2+^** formed depends on both the electronic structure of the photoswitch and the scan rate. While at slow scan rates (10 mV s^−1^) the conversion is quantitative for **sDTE_66_-Me**, it can still be observed at the fast scan rates (1 V s^−1^). Notably, for phenyl-substituted sDTEs the extent of **FP^2+^**-formation is lower, and for the donor-substituted ***C-*sDTE_66_-PhOMe** the initial absorption spectrum after a complete redox cycle is fully recovered, even at low scan rates (for a comparison of full redox cycles of ***C-*sDTE_66_-Me** and ***C-*sDTE_66_-PhOMe** see Figure S30, Supporting Information File 1). This observation confirms the enhanced stabilization of the ***C^2+^*** cation by electron-donating groups [8]. 3) Upon reduction of **FP^2+^**, both **FP** as well as ***C*** are formed. UPLC analysis of a preparative scale oxidation and subsequent reduction of ***Z-*sDTE_66_-Me** as well as ***C-*sDTE_66_-Me** (Figure S41 and Figure S42, Supporting Information File 1) showed mainly the closed isomer after the reduction step. However, in these experiments an insoluble off-red film deposited on the platinum electrode that resisted further analysis (for a photograph, see Figure S41d, Supporting Information File 1). 4) The absorption spectrum of **FP** is red-shifted compared to the two open isomers yet blue-shifted compared to the closed isomer (Figure S29b, Supporting Information File 1). 5) Upon reduction, a species analogous to **FP** was not observed (vide infra and Figure S43, Supporting Information File 1).

**Scheme 3:**
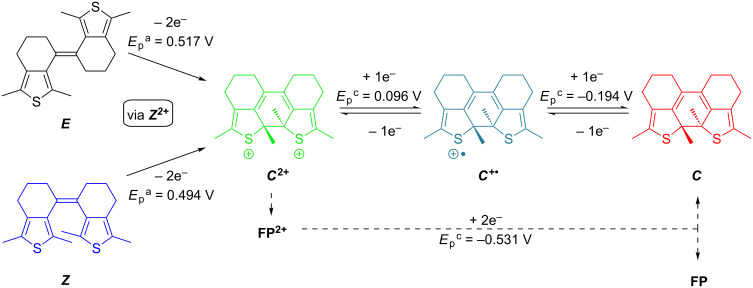
Proposed mechanism for the oxidative cyclization of **sDTE_66_-Me**. Upon two-fold oxidation, both open isomers cyclize to the dication ***C^2+^***. This dication can reversibly be reduced to the closed isomer ***C*** in two consecutive steps. Upon low sweep rates, an unidentified follow-up product **FP** forms that at least partially can be reduced to ***C***.

Considering all these findings, a mechanism for the oxidative cyclization of **sDTE_66_-Me** (Scheme 3) can be derived: Starting from either open isomer fast isomerization to the same dication ***C^2+^*** takes place upon oxidation. Both, the isomerization of the double bond of the *E-*isomer as well as the cyclization reaction are instantaneous on the timescale of the experiment. The closed dication ***C*^2+^** can be reduced stepwise to its neutral form. In addition, when ***C*^2+^** is not stabilized by donor substituents it undergoes a subsequent reaction to the structurally yet unidentified follow-up product **FP^2+^**, which upon reduction, at least partially, transforms into the charge-neutral closed isomer ***C***. Note that during oxidative cyclization the characteristic absorption of the cation radical ***C^+•^*** at 731 nm is not observed (Figure 2a and b), suggesting a concerted two-electron oxidation of the open isomers and thermal cyclization in the dicationic state (vide infra).

### Influence of ring size and substitution

**Figure 3:**
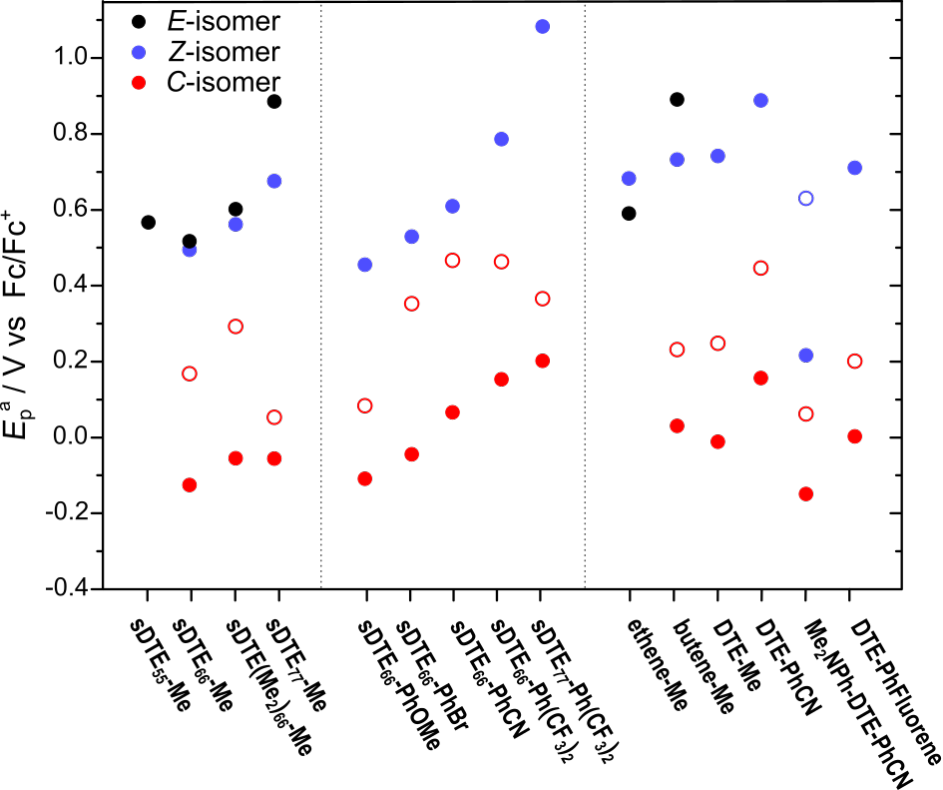
Anodic peak potentials (*E*_p_^a^) of sDTEs and reference compounds in MeCN. Solid circles refer to the first oxidation potential, hollow circles to the second oxidation potential (if available). For detailed data, see Table S1, for all respective cyclovoltammograms, see Figure S10 to Figure S27 in Supporting Information File 1.

To correlate structural parameters with the observed electrochemical behavior, sDTEs with other ring sizes (**sDTE_55_-Me** and **sDTE_77_-R**) and derivatives bearing peripheral phenyl substituents with different electronic properties (-OMe, -Br, -CN, (-CF_3_)_2_) as well as various reference compounds were examined. The oxidation potentials for the investigated compounds (see Scheme 2) are summarized in Figure 3 and Table S1 (Supporting Information File 1).

For most ring-open compounds investigated, the *E-*isomer displayed a higher or at least equal oxidation potential when compared to the *Z-*isomer. This difference for both open isomers is rather surprising in view of their very similar absorption maxima in first approximation reflecting the HOMO–LUMO gap. The sole exception to this trend is the only derivative with an H-substituted double bond **ethene-Me**, which exhibit the expected behavior of a less facile oxidation of the *Z-*isomer due to its somewhat twisted π-system leading to less pronounced π-conjugation and lowering the HOMO level. Even more surprising is the comparison of methyl-substituted derivatives of different ring sizes. Instead of the expected ease of oxidation with an increasing number of carbons due to their donating inductive (+I) effect, the exact opposite trend was observed with **sDTE_77_-Me** being most difficult to oxidize. In the group of sDTE_66_-R derivatives, however, the influence of the substituent is in accordance with its electron-donating or electron-withdrawing ability.

The closed isomers are, in general, much easier to oxidize as the open isomers, even in cases such as **sDTE_77_-Me**, in line with the largely reduced HOMO–LUMO gap of the colored closed isomers implying an energetically higher and thus more accessible HOMO level. The first and second oxidation potential are shifted depending on the electron-donating or electron-withdrawing character of the attached substituents, similarly to what is known for normal DAEs [40–41]. The differences between the first and second oxidation wave seem to depend on the nature of the substituents with donors reducing the gap, presumably by more efficient stabilization of the dication, and on conformational rigidity dictating the extent of π-conjugation between both hemispheres.

Similar to the model compound **sDTE_66_-Me**, all available sDTE_66_ derivatives as well as both *Z*-configured sDTE_77_ derivatives undergo electrocyclization upon oxidation. This also holds true for all cyclopentene-bridged DTE derivatives. However, in **butene-Me** the formation of **FP^2+^** is the predominant reaction pathway even at high scan rates, while for ***E-*sDTE_55_-Me** as well as both isomers of **ethene-Me** no characteristic cathodic waves of the closed isomers were observed. For ***E-*sDTE_77_-Me** neither oxidative cyclization nor formation of **FP^2+^** was found despite its irreversible oxidation wave.

### Cyclization by cathodic reduction

Except for rare examples of methylpyridinium substituted DTEs [27–28] and dithiazolylethenes [29], ring closure under reductive conditions has not been reported for DAEs. For most structures the reduction potential of the open isomer is too negative to be reached within the redox window determined by the electrolyte. Thus, a strongly electron-deficient substituent such as pyridinium is necessary to shift the reduction potential to accessible values. However, not every electron-withdrawing group appears to be suited to induce cathodic cyclization [40,42].

**Figure 4:**
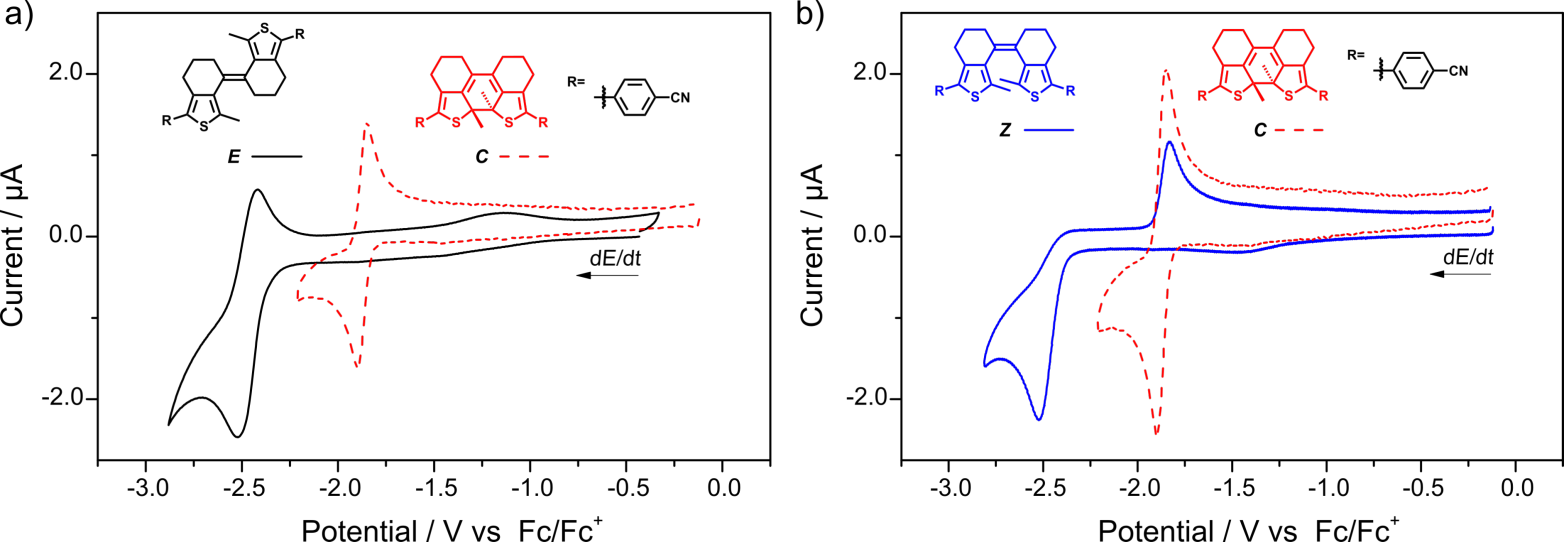
Cyclic voltammograms of **sDTE_66_-PhCN**. The reduction of a) ***E-*sDTE_66_-PhCN** (black line) is reversible, whereas the reduction of b) ***Z-*sDTE_66_-PhCN** (blue line) yields the closed isomer, indicated by the characteristic oxidation wave of ***C-*sDTE_66_-PhCN** (red dashed line), generated photochemically. Experiments in a) DMF with 0.1 M Bu_4_NPF_6_, *c* = 5∙10^−4^ M, *dE*/*dt* = 1 V s^−1^ and b) MeCN with 0.1 M Bu_4_NPF_6_, *c* = 5∙10^−4^ M, *dE*/*dt* = 1 V s^−1^.

In the case of the electron-deficient benzonitrile derivative **sDTE_66_-PhCN** the reduction potential could be reached and for the *Z-*isomer an irreversible two-electron reduction wave at *E*_p_^c^ = −2.526 V was detected (Figure 4). Upon re-oxidation, a new peak matching that of the photochemically generated closed isomer, determined in a separate experiment, was observed. Likewise, in a second cycle (Figure S24, Supporting Information File 1), the reduction wave of the closed isomer at *E*_p_^c^ = −1.920 V appeared, clearly indicating reductive cyclization. The reductive formation of ***C-*sDTE_66_-PhCN** was unequivocally proven by a preparative electrolysis of ***Z-*sDTE_66_-PhCN** and subsequent product analysis, showing a closed to *Z-*isomer ratio of 69:31 (Figure S43, Supporting Information File 1). Interestingly and in strong contrast to the *Z-*isomer, for the *E-*isomer a reversible reduction wave was observed and no cyclization product was formed (Figure 4a). Note that, however, both open isomers undergo oxidative cyclization (Figure S19b and Figure S20b, Supporting Information File 1).

To investigate the generality of reductive cyclization mediated by cyano groups, we subjected the cyclopentene-bridged DTE with benzonitrile substituents (**DTE-PhCN**) to these conditions. The electrochemistry of this compound [8] and its hexafluorocyclopentene derivative [43] have already been investigated, but its behavior under reductive conditions has not been discussed. Indeed, an irreversible reduction wave at *E*_p_^c^ = −2.436 V was found, giving rise to an oxidation wave corresponding to the closed isomer (Figure S23, Supporting Information File 1). Once formed, the closed isomers of both nitrile-substituted compounds, i.e., **sDTE_66_-PhCN** and **DTE-PhCN**, can be reversibly reduced and oxidized (Figure S20 and Figure S23, Supporting Information File 1). The two-electron reduction of the closed isomer was found to occur in a stepwise manner as indicated by SEC of ***C*-DTE-PhCN** clearly showing an intermediate radical anionic species ***C^−•^*** (Figure S40, Supporting Information File 1).

**Figure 5:**
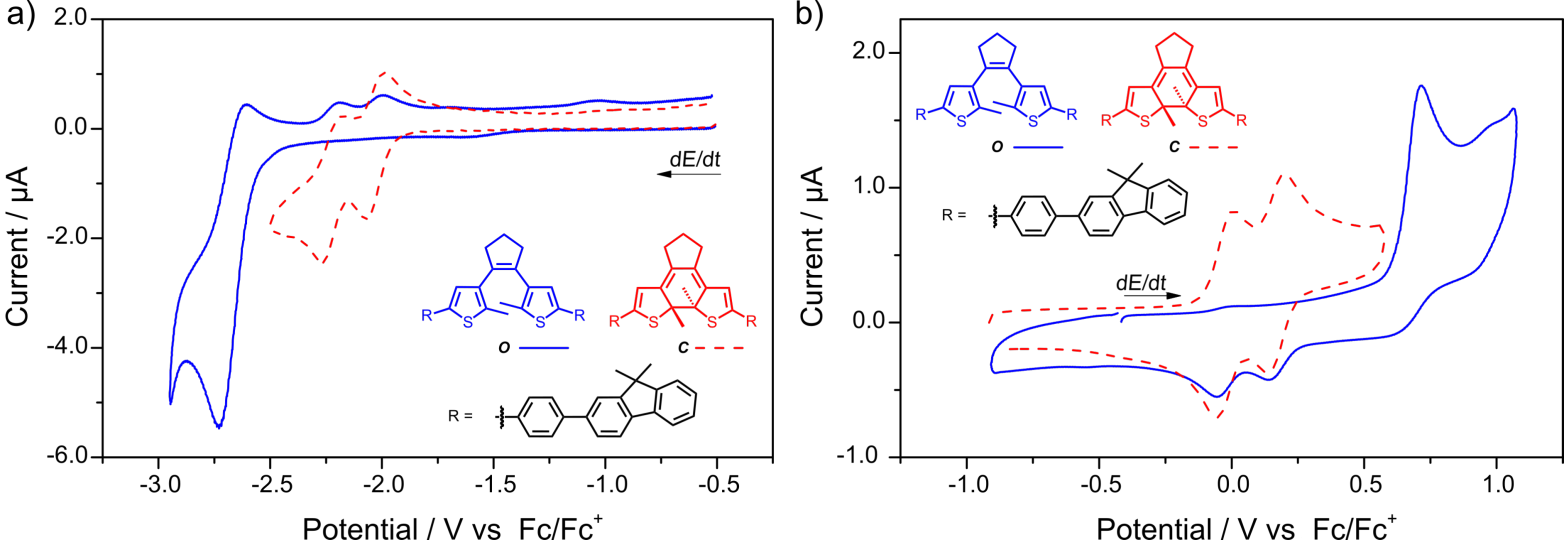
Cyclic voltammogram of **DTE-PhFluorene**. The ring-closed isomer (red dashed line) is formed both under a) reductive and b) oxidative conditions from ***O*-DTE-PhFluorene** (blue line), as shown by the emerging characteristic oxidation and reduction waves, respectively. Experiments in a) DMF with 0.1 M Bu_4_NPF_6_, *c* = 1∙10^−3^ M, *dE*/*dt* = 1 V s^−1^ and b) DCM with 0.2 M Bu_4_NPF_6_ *c* = 7∙10^−4^ M, *dE*/*dt* = 100 mV s^−1^. For two consecutive oxidation and reduction cycles, see Figure S25 in Supporting Information File 1.

Moreover, the phenomenon of reductive cyclization appears not to be restricted to DTEs with strongly electron-withdrawing groups. We found that the electronic effects of an extended π-system lead to similar results. As such, the bis(4-(9,9-dimethyl-9*H*-fluoren-2-yl)phenyl)-substituted **DTE-PhFluorene** undergoes reductive as well as oxidative cyclization (Figure 5), a phenomenon that we have recently also observed for reductively and oxidatively induced azobenzene *Z*→*E* isomerization [44–45].

### Mechanism of the electrochemical isomerization

In the literature, either mono- [7,18,22–23,46] or bis-oxidized [9–11] open DAEs have been reported and discussed [8,47] as the key intermediate undergoing thermal cyclization or cycloreversion, typically in the context of so-called “ECE” and “EEC” mechanisms, respectively [48]. To contribute to this discussion, nonsymmetrical DAEs bearing two electronically distinct aryl moieties (CF_3_- and Me-thiazole) [5] or thiophenes possessing donor and acceptor substituents (-Ph/-PhOMe, -Ph(CF_3_)_2_/-PhNMe_2_, and -Ph(CF_3_)_2_/-PhOMe) have been investigated [8,40]. However, sufficient separation of the oxidation waves in order to assure a fully stepwise oxidation process is difficult to achieve and could only be realized in the case of modified thiazoles [5] or using the strongly donating -PhNMe_2_ substituent [40]. In these compounds, the first oxidation wave is fully reversible and anodic cyclization occurs only after the second step, forming the open dication (EEC mechanism).

**Figure 6:**
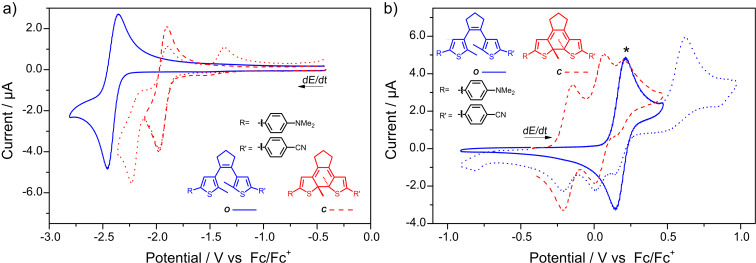
Cyclic voltammograms of **Me_2_NPh-DTE-PhCN** displaying separated one-electron anodic and cathodic waves. a) Reversible first one-electron reduction (blue line) of ***O-*Me_2_NPh-DTE-PhCN**. The second reduction potential cannot be accessed. First (red dashed line) and second one-electron reduction (red dotted line) of ***C-*Me_2_NPh-DTE-PhCN** are shown for comparison. b) Oxidative cyclization of ***O-*Me_2_NPh-DTE-PhCN** occurs only at the second oxidation/reduction step (blue dotted line), whereas the first step (blue line) is reversible. The oxidation waves of the closed isomer (red dashed line) are shown for comparison. The asterisk marks the oxidation wave of residual open isomer after irradiation. Experiments in MeCN with 0.1 M Bu_4_NPF_6_, *c* = 1∙10^−3^ M, *dE*/*dt* = 1 V s^−1^.

By combining strongly electron-donating and electron-withdrawing substituents within one molecule (**Me_2_NPh-DTE-PhCN**), we could access an open isomer showing two separated one-electron waves upon both oxidation and reduction (Figure 6). As in the examples above, the first oxidation wave of ***O*-Me_2_NPh-DTE-PhCN** is fully reversible and anodic cyclization occurs only from the open dication (Figure 6b). In addition, also the first reduction wave is fully reversible (Figure 6a). However, because the second reduction potential was not accessible, no cyclization was observed. It is noteworthy that the reduction of ***O*-Me_2_NPh-DTE-PhCN** and ***O*-DTE-PhCN** occur at very similar potentials (−2.457 V and −2.436 V, respectively), thus indicating that both hemispheres in open DTEs are only very weakly conjugated.

**Scheme 4:**
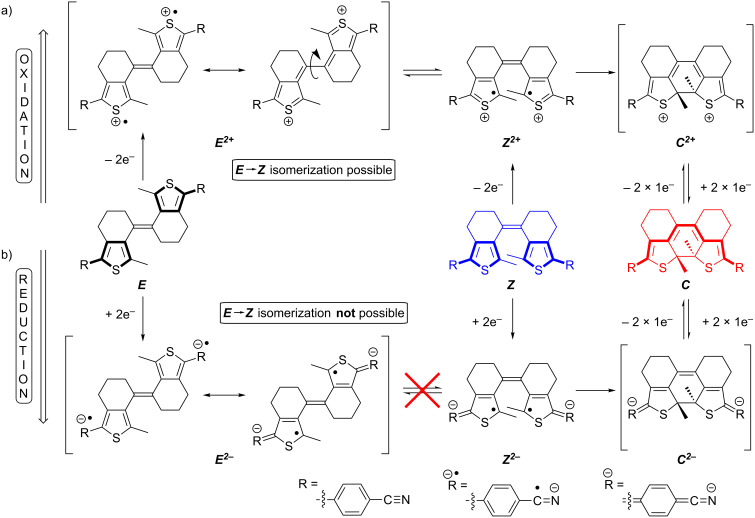
Proposed mechanism to explain the observed selectivity of anodic and cathodic cyclization in sDTE_66_ derivatives: a) Upon two-fold oxidation, an equilibration between the two dications of the *E-* and *Z-*isomers is possible due to rotation about the formed central single bond. Once formed, ***Z^2+^*** is trapped as ***C^2+^***. b) In contrast, bond rotation is not possible for the dianion ***E^2−^*** and as a consequence, reductive cyclization is only possible from ***Z^2−^***.

From the fact that the radical anion does not undergo cyclization we conclude that a concerted EEC mechanism is valid for both oxidative and reductive cyclization in our compounds (Scheme 4). There is, however, a marked difference between the oxidative and reductive pathway: In the dicationic state a resonance structure exists in which the central double bond is resolved and thus bond rotation is allowed. This is reflected by the fact that both the *E-* and the *Z-*isomers of sDTEs are able to undergo oxidative cyclization. In contrast, in the dianionic state formal cross-conjugation between the thiophene moieties and the central double bond persists and no rotation takes place. Thus, only the dianion of the *Z-*isomer undergoes cyclization while the reduction of the *E-*isomer is reversible. This behavior differs strongly from that of stilbene, in which reduction occurs at the double bond and causes an equilibration of the double bond isomers in favor of the *E-*stilbene [49].

## Conclusion

We have investigated the electrochemical behavior of sDTEs, a new family of photoswitches possessing three switching states (*E*/*Z*/*C*). The oxidation potentials of a series of compounds with either 5-, 6-, or 7-membered rings attached to the central exocyclic double bond as well as the electronic influence of substituents were compared. We found that in particular the derivatives with 6-membered rings undergo efficient cyclization upon two-electron oxidation. Notably, both *E-* and *Z-*isomers undergo 6π-electrocyclization because rotation about the central bond is possible in the dication. Furthermore, we discovered three examples undergoing cyclization upon two-electron reduction that so far has only rarely been observed. Remarkably, only the *Z-*isomer ring-closes under reductive conditions, whereas the *E-*isomer is reduced reversibly. In addition, the investigation of a nonsymmetrically substituted DTE showed that a two-fold change of the oxidation state is necessary to achieve cyclization, via both the oxidative and reductive pathway.

## Experimental

A detailed description of the experimental conditions and applied analytical methods, including synthesis, photochemistry, and electrochemistry, can be found in Supporting Information File 1.

## Supporting Information

File 1 Experimental part.

## References

[1] Irie M, Mohri M (1988). J Org Chem.

[2] Nakamura S, Irie M (1988). J Org Chem.

[3] Irie M (2000). Chem Rev.

[4] Irie M, Fukaminato T, Matsuda K, Kobatake S (2014). Chem Rev.

[5] Herder M, Utecht M, Manicke N, Grubert L, Pätzel M, Saalfrank P, Hecht S (2013). Chem Sci.

[6] Peters A, Branda N R (2003). Chem Commun.

[7] Browne W R, de Jong J J D, Kudernac T, Walko M, Lucas L N, Uchida K, van Esch J H, Feringa B L (2005). Chem – Eur J.

[8] Browne W R, de Jong J J D, Kudernac T, Walko M, Lucas L N, Uchida K, van Esch J H, Feringa B L (2005). Chem – Eur J.

[9] Liu Y, Lagrost C, Costuas K, Tchouar N, Le Bozec H, Rigaut S (2008). Chem Commun.

[10] He B, Wenger O S (2011). J Am Chem Soc.

[11] Staykov A, Areephong J, Browne W R, Feringa B L, Yoshizawa K (2011). ACS Nano.

[12] Harvey E C, Areephong J, Cafolla A A, Long C, Browne W R, Feringa B L, Pryce M T (2014). Organometallics.

[13] Meng F, Hervault Y-M, Shao Q, Hu B, Norel L, Rigaut S, Chen X (2014). Nat Commun.

[14] Koshido T, Kawai T, Yoshino K (1995). J Phys Chem.

[15] Peters A, Branda N R (2003). J Am Chem Soc.

[16] Zhou X-H, Zhang F-S, Yuan P, Sun F, Pu S-Z, Zhao F-Q, Tung C-H (2004). Chem Lett.

[17] Nakashima T, Kajiki Y, Fukumoto S, Taguchi M, Nagao S, Hirota S, Kawai T (2012). J Am Chem Soc.

[18] Lee S, You Y, Ohkubo K, Fukuzumi S, Nam W (2012). Org Lett.

[19] Massaad J, Micheau J-C, Coudret C, Serpentini C L, Guirado G (2013). Chem – Eur J.

[20] Lee S, You Y, Ohkubo K, Fukuzumi S, Nam W (2014). Chem Sci.

[21] Calupitan J P, Nakashima T, Hashimoto Y, Kawai T (2016). Chem – Eur J.

[22] Moriyama Y, Matsuda K, Tanifuji N, Irie S, Irie M (2005). Org Lett.

[23] Guirado G, Coudret C, Hliwa M, Launay J-P (2005). J Phys Chem B.

[24] Coudret C, Guirado G, Hortholary C, Launay J-P, Battaglini N, Klein H, Dumas P (2005). Mol Cryst Liq Cryst.

[25] Yuan N, Zhang Z, Wang X, Wang X (2015). Chem Commun.

[26] Zhang Z-X, Wang P-X, Bai F-Q, Kong C-P, Zhang H-X (2017). Phys Chem Chem Phys.

[27] Gorodetsky B, Samachetty H D, Donkers R L, Workentin M S, Branda N R (2004). Angew Chem.

[28] Gorodetsky B, Branda N R (2007). Adv Funct Mater.

[29] Léaustic A, Anxolabéhère-Mallart E, Maurel F, Midelton S, Guillot R, Métivier R, Nakatani K, Yu P (2011). Chem – Eur J.

[30] Ogawa K, Suzuki H, Futakami M (1988). J Chem Soc, Perkin Trans 2.

[31] Oelgemöller M, Brem B, Frank R, Schneider S, Lenoir D, Hertkorn N, Origane Y, Lemmen P, Lex J, Inoue Y (2002). J Chem Soc, Perkin Trans 2.

[32] Oelgemöller M, Frank R, Lemmen P, Lenoir D, Lex J, Inoue Y (2012). Tetrahedron.

[R33] 33Kleinwächter, M.; Teichmann, E.; Schwarz, J.; Hecht, S. *manuscript in preparation.*

[34] Lewis F D, Petisce J R, Oxman J D, Nepras M J (1985). J Am Chem Soc.

[35] Majima T, Tojo S, Ishida A, Takamuku S (1996). J Org Chem.

[36] Bragadin M, Cescon P, Berlin A, Sannicolò F (1987). Makromol Chem.

[37] Onoda M, Iwasa T, Kawai T, Yoshino K (1991). J Phys Soc Jpn.

[38] Benincori T, Brenna E, Sannicolò F, Trimarco L, Schiavon G, Zecchin S, Zotti G (1996). Macromol Chem Phys.

[39] Irie M, Lifka T, Uchida K, Kobatake S, Shindo Y (1999). Chem Commun.

[40] Herder M, Schmidt B M, Grubert L, Pätzel M, Schwarz J, Hecht S (2015). J Am Chem Soc.

[41] Herder M, Eisenreich F, Bonasera A, Grafl A, Grubert L, Pätzel M, Schwarz J, Hecht S (2017). Chem – Eur J.

[42] Berberich M, Würthner F (2013). Asian J Org Chem.

[43] Liu G, Pu S Z, Zheng C H, Le Z G, Luo M B (2007). Phys Scr.

[44] Goulet-Hanssens A, Utecht M, Mutruc D, Titov E, Schwarz J, Grubert L, Bléger D, Saalfrank P, Hecht S (2017). J Am Chem Soc.

[45] Goulet-Hanssens A, Rietze C, Titov E, Abdullahu L, Grubert L, Saalfrank P, Hecht S (2018). Chem.

[46] Matsuda K, Yokojima S, Moriyama Y, Nakamura S, Irie M (2006). Chem Lett.

[47] Logtenberg H, Browne W R (2013). Org Biomol Chem.

[48] Testa A C, Reinmuth W H (1961). Anal Chem.

[49] Abdul-Rahim O, Simonov A N, Boas J F, Rüther T, Collins D J, Perlmutter P, Bond A M (2014). J Phys Chem B.

