# Supplementary material for: Oxidative and reductive cyclization in stiff dithienylethenes
Source: Beilstein J Org Chem. 2018 Nov 9;14:2812–21. doi: 10.3762/bjoc.14.259 (PMC6244239; doi:10.3762/bjoc.14.259)
Supplement: File 1 — Experimental part. [file Beilstein_J_Org_Chem-14-2812-s001.pdf]

Supporting Information

for

Oxidative and reductive cyclization in stiff dithienylethenes

Michael Kleinwächter, Ellen Teichmann, Lutz Grubert, Martin Herder and Stefan Hecht*

Address: Department of Chemistry & IRIS Adlershof, Humboldt-Universität zu Berlin, Brook-Taylor-Straße 2, 12489 Berlin, Germany

Email: Stefan Hecht - sh@chemie.hu-berlin.de

*Corresponding author

Experimental part

General methods

1,2-Bis(5-chloro-2-methylthien-3-yl)cyclopent-1-ene [1], 1,2-bis(5-(4-bromophenyl)-2-methyl-thien-3-yl)cyclopent-1-ene [2] and 2-(9,9-dimethyl-9*H*-fluoren-2-yl)-4,4,5,5-tetramethyl-1,3,2-dioxaborolane [3] were prepared according to literature procedures. All solvents and other starting materials were commercial and used as received. Dry solvents were obtained employing an Innovative Technologies solvent purification system. THF was degassed and further dried by storing over activated molecular sieves (4 Å). All reactions involving light sensitive compounds were carried out in the dark under red light. The NMR spectra were recorded on a 500 MHz Bruker AVANCE II 500 spectrometer at 25 °C, if not mentioned otherwise, with the spectra referenced to the residual solvent signal (^{1}H: δ ($CHCl_3$) = 7.26 ppm, δ (THF-d_7) = 3.58 ppm and ^{13}C: δ ($CHCl_3$) = 77.16 ppm, *δ* (THF-d_7) = 67.57 ppm). UPLC–MS was performed on a Waters UPLC Acquity equipped with a Waters LCT Premier XE Mass Detector for UPLC–HRMS, with Waters Alliance systems (consisting of a Waters Separations Module 2695, a Waters Diode Array Detector 996 and a Waters Mass Detector ZQ 2000). Preparative GPC was performed on a LC-9210NEXT preparative recycling GPC (Japan Analytical Industry). The ESI+/− values are obtained from UPLC chromatograms.

Photochemical setup

The irradiation experiments were carried out in degassed spectroscopy grade solvents (HPLC-MS grade for MeCN). The analytical UV–vis spectroscopy was performed on a Cary 50 and Cary 60 UV–vis spectrophotometer equipped with a thermostated cell holder at 25 °C. The irradiation was performed in a quartz cuvette using either a 500 W mercury lamp coupled with a grating monochromator or a 1000 W high pressure Xenon lamp with optical band pass or cut-off filters.

Electrochemical setup

Cyclic voltammetry was performed using a PG310 USB (HEKA Elektronik) potentiostat interfaced to a PC with PotMaster v2x43 (HEKA Elektronik) software for data evaluation. A three-electrode configuration was used, contained in a non-divided cell consisting of a platinum disc (*d* = 1 mm) as working electrode, a platinum plate as counter-electrode, and a saturated calomel electrode (SCE) with an agar-agar-plug in a Luggin capillary with a diaphragm as reference electrode. Measurements were carried out in $1\cdot10^{-3}$ M solutions in acetonitrile (HPLC-grade, dried over calcium hydride and distilled) containing 0.1 M Bu_4NPF_6 using a scan rate of dE/dt = 1 V s^{-1}, in DCM (HPLC-grade, dried over calcium hydride and distilled) containing 0.2 M Bu_4NPF_6 and a scan rate of dE/dt = 0.1 V s^{-1} or in DMF (Sigma-Aldrich, anhydrous 99.8%) containing 0.1 M Bu_4NPF_6 and a scan rate of dE/dt = 1 V s^{-1}. The data is given in reference to the ferrocene redox couple (Fc/Fc^{+}), which was used as external standard. Cyclic voltammograms of ring-closed isomers of diarylethenes were obtained by irradiation of the electrochemical cell using a standard laboratory UV-lamp equipped with a 313 nm UV-tube (Vilber Lourmat, 6 W).

Spectro-electrochemistry was performed in a SEC-C Thin Layer Quartz Glass Spectro-electrochemical cell, 0.5 or 1.0 mm optical path length, with platinum mesh electrode as a working electrode, counter electrode: platinum wire, reference electrode: non aqueous reference electrode Ag/Ag^{+} (0.01 M $AgNO_3$ in 0.1 M Bu_4NPF_6 acetonitrile), ALS Co., Ltd (Tokyo, Japan) spectrometer: AvaSpec-2048x14 with AvaLight-DH-S-BAL, and AvaSoft 7.7.2, Avantes

(Apeldoorn, Netherlands) Potentiostat: PGSTAT 128N, Deutsche Metrohm GmbH & Co. KG (Filderstadt, Germany) and software: NOVA 1.10. Scan rate for all solvents dE/dt = 10 mV s^{-1}.

Preparative electrolysis was performed in a double H cell equipped with a platinum net.

Synthesis

1-(5-(4-*N,N*-Dimethylaminophenyl)-2-methylthien-3-yl)-2-(5-chloro-2-methylthien-3-yl)cyclopent-1-ene (Me_2NPh-DTE-Cl)

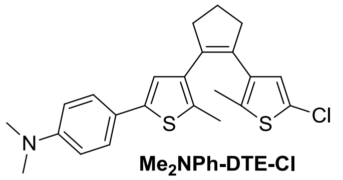

In analogy to a literature procedure [1], 1,2-bis(5-chloro-2-methylthien-3-yl)cyclopent-1-ene [1] (0.659 g, 2.00 mmol, 1.0 equiv) was dissolved in 20 mL of anhydrous THF under argon atmosphere. The resulting solution was cooled to −78 °C, *n*-butyllithium (2.3 M in cyclohexane, 1.30 mL, 3.00 mmol, 1.5 equiv) was added dropwise and the solution was stirred at that temperature for 30 min. Then tri(isobutyl) borate (0.752 g, 0.923 mL, 4.00 mmol, 2.0 equiv) was added and the solution was stirred for 1 h while gently warming to room temperature. In the meantime, 4-bromo-*N,N*-dimethylaniline (0.480 g, 2.40 mmol, 1.2 equiv) was dissolved in 20 mL of degassed THF and $Pd(PPh_3)_4$ (0.231 g, 0.20 mmol, 0.1 equiv) was added at once resulting in a bright yellow solution which was stirred for 15 min. An aqueous solution of Na_2CO_3 (2 M, 4.00 mL, 8.00 mmol, 4.0 equiv), 5 droplets of ethylene glycol and the previously prepared solution of the borylated species were added and the resulting two-phase system was heated to 65 °C for 14 h.

After cooling to room temperature, the reaction mixture was diluted with 100 mL of water and extracted with 3 × 50 mL of ethyl acetate. The combined organic layers were washed with 100 mL of water and saturated NaCl solution, dried over $MgSO_4$ and concentrated under reduced pressure. Purification by column chromatography (ethyl acetate / cyclohexane) afforded the title compound (0.678 g, 1.64 mmol, 82% yield) as a brownish solid.

^{1}H-NMR (500 MHz, $CDCl_3$): δ [ppm] = 7.38 (d, $^3J_{H,H}$ = 8.9 Hz, 2H, CH_{ar}), 6.82 (s, 1H, CH_{tph}), 6.70 (d, $^3J_{H,H}$ = 8.9 Hz, 2H, CH_{ar}), 6.63 (s, 1H, CH_{tph}), 2.96 (s, 6H, NCH_3), 2.83 - 2.71 (m, 4H, CH_2), 2.00 - 1.92 (m, 2H, CH_2), 1.97 (s, 3H, CH_3), 1.88 (s, 3H, CH_3).

^{13}C-NMR (126 MHz, $CDCl_3$): δ [ppm] = 149.9, 140.7, 136.2, 135.8, 135.4, 133.4, 132.5, 131.8, 127.0, 125.0, 123.2, 121.7, 114.2, 112.8, 40.7, 38.6, 38.5, 23.1, 14.5, 14.3.

HR-MS (ESI^+) m/z = 414.112 (calcd. 414.111 for $[C_{23}H_{24}ClNS_2+H]^+$).

1-(5-(4-*N,N*-Dimethylaminophenyl)-2-methylthien-3-yl)-2-(5-(4-cyanophenyl)-2-methylthien-3-yl)cyclopent-1-ene (Me_2NPh-DTE-PhCN)

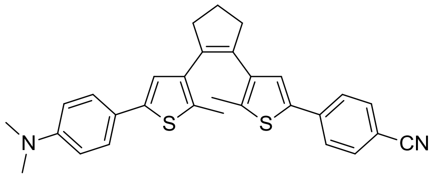

Me_2NPh-DTE-PhCN

The title compound was synthesized following the procedure described for preparation of **Me_2NPh-DTE-Cl** using compound **Me_2NPh-DTE-Cl** (166 mg, 0.400 mmol, 1.0 equiv) in 10 mL of anhydrous THF, *n*-butyllithium (2.3 M in cyclohexane, 0.435 mL, 1.00 mmol, 2.5 equiv), tri(isobutyl) borate (0.188 g, 0.231 mL, 1.00 mmol, 2.5 equiv), 4-bromobenzonitrile (87.0 mg, 0.480 mmol, 1.2 equiv) in 10 mL of THF, $Pd(PPh_3)_4$ (46.0 mg, 0.040 mmol, 0.1 equiv) and an aqueous solution of Na_2CO_3 (2 M, 0.800 mL, 1.60 mmol, 4.0 equiv). The reaction was performed at 65 °C for 16 h. Purification by column chromatography (ethyl acetate/cyclohexane) followed by GPC afforded the title compound (97.0 mg, 0.202 mmol, 50% yield) as greenish oil which was lyophilized from benzene to give a greenish powder.

^{1}H-NMR (500 MHz, $CDCl_3$, Figure S1): δ [ppm] = 7.59 (d, $^3J_{H,H}$ = 8.7 Hz, 2H, CH_{ar}), 7.54 (d, $^3J_{H,H}$ = 8.7 Hz, 2H, CH_{ar}), 7.38 (d, $^3J_{H,H}$ = 8.9 Hz, 2H, CH_{ar}), 7.13 (s, 1H, CH_{tph}), 6.86 (s, 1H, CH_{tph}), 6.70 (d, $^3J_{H,H}$ = 8.9 Hz, 2H, CH_{ar}), 2.96 (s, 6H, NCH_3), 2.87 - 2.80 (m, 4H, CH_2), 2.12 - 2.05 (m, 2H, CH_2), 2.06 (s, 3H, CH_3), 1.96 (s, 3H, CH_3).

^{13}C-NMR (126 MHz, $CDCl_3$, Figure S2): δ [ppm] = 149.9, 140.8, 139.0, 137.6, 137.4, 137.3, 136.3, 136.0, 133.7, 132.8, 132.4, 126.5, 126.4, 125.5, 123.2, 121.7, 119.2, 112.7, 109.8, 40.7, 38.6, 38.5, 23.2, 14.8, 14.5.

HR-MS (ESI^+) *m/z* = 481.178 (calcd. 481.177 for $[C_{30}H_{28}N_2S_2+H]^+$).

1,2-Bis(5-(4-cyanophenyl)-2-methylthien-3-yl)cyclopent-1-ene (DTE-PhCN)

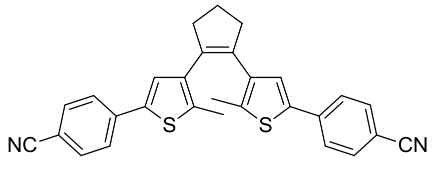

DTE-PhCN

The title compound was synthesized following the procedure described for **Me_2NPh-DTE-Cl** using 1,2-bis(5-chloro-2-methylthien-3-yl)cyclopent-1-ene [1] (0.329 g, 1.00 mmol, 1.0 equiv) in 10 mL of anhydrous THF, *n*-butyllithium (2.3 M in cyclohexane, 1.74 mL, 4.00 mmol, 4.0 equiv), tri(isobutyl) borate (0.752 g, 0.923 mL, 4.00 mmol, 4.0 equiv), 4-bromobenzonitrile (0.546 g, 3.00 mmol, 3.0 equiv) in 10 mL of THF, $Pd(PPh_3)_4$ (0.116 g, 0.10 mmol, 0.1 equiv) and an aqueous solution of Na_2CO_3 (2 M, 2.00 mL, 4.00 mmol, 4.0 equiv). The reaction was performed at 65°C for 17 h. Purification by column chromatography (ethyl acetate/cyclohexane), recrystallization from chloroform/ethanol and GPC afforded the title compound (0.181 g, 0.392 mmol, 39% yield) as a white solid.

^{1}H-NMR (500 MHz, THF-d$_8$, Figure S3): δ [ppm] = 7.70 - 7.65 (m, 8H, CH_{ar}), 7.36 (s, 2H, CH_{tph}), 2.93 - 2.85 (m, 4H, CH_2), 2.14 - 2.06 (m, 2H, CH_2), 1.99 (s, 6H, CH_3).
^{13}C-NMR (126 MHz, THF-d$_8$, Figure S4): δ [ppm] = 139.5, 139.0, 138.5, 137.8, 136.0, 133.7, 127.4, 126.4, 119.2, 111.6, 39.4, 24.0, 14.8.
HR-MS (ESI$^+$) *m/z* = 463.124 (calcd. 463.130 for $[C_{29}H_{22}N_2S_2+H]^+$).

1,2-Bis(5-(4-(9,9-dimethyl-9*H*-fluoren-2-yl)phenyl)-2-methylthien-3-yl)cyclopent-1-ene (DTE-PhFluorene)

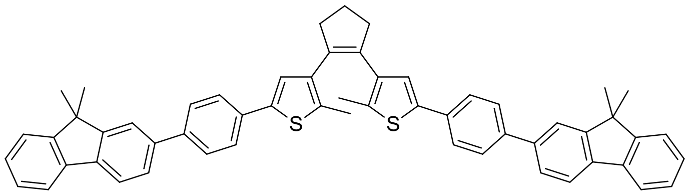

DTE-PhFluorene

1,2-Bis(5-(4-bromophenyl)-2-methylthien-3-yl)cyclopent-1-ene [2] (915 mg, 1.61 mmol, 1.0 equiv) and 2-(9,9-dimethyl-9*H*-fluoren-2-yl)-4,4,5,5-tetramethyl-1,3,2-dioxaborolane [3] (1.079 g, 3.37 mmol, 2.1 equiv) were dissolved in 30 mL of toluene and the mixture was degassed by bubbling with argon for 10 min. A degassed solution of Na_2CO_3 (2 M, 7.5 mL) as well as $PdCl_2(PPh_3)_2$ (113 mg, 0.16 mmol, 0.1 equiv) were added and the mixture was stirred at 110 °C for 15 h. After cooling to room temperature, the mixture was diluted with ethyl acetate and was washed with brine (3×). The organic phase was dried over $MgSO_4$ and evaporated. Purification by column chromatography (petrol ether/methylene chloride 4:1) afforded the title compound (270 mg, 0.34 mmol, 21%) as a white solid.
^{1}H-NMR (300 MHz, $CDCl_3$, Figure S5): *δ* (ppm) = 7.81 – 7.72 (m, 4 H), 7.69 – 7.56 (m, 12 H), 7.49 – 7.43 (m, 2 H), 7.39 – 7.30 (m, 4 H), 7.13 (s, 2 H), 2.90 (t, $^{3}J_{H,H}$ = 7.4 Hz, 4 H), 2.13 (pent, $^{3}J_{H,H}$ = 7.4 Hz, 2 H), 2.06 (s, 6 H), 1.54 (s, 12 H).
^{13}C-NMR (75.5 MHz, $CDCl_3$, Figure S6): *δ* (ppm) = 154.4, 154.0, 140.1, 139.8, 139.4, 138.9, 138.6, 136.9, 134.82, 134.75, 133.5, 127.5 (*C*H), 127.4 (*C*H), 127.1 (*C*H), 126.0 (*C*H), 125.7 (*C*H), 124.2 (*C*H), 122.7 (*C*H), 121.1 (*C*H), 120.4 (*C*H), 120.1 (*C*H), 47.0, 38.6 (CH_2), 27.3 (CH_3), 23.2 (CH_2), 14.6 (CH_3).
HRMS (ESI+): 796.319 (calcd. 796.319 for $[C_{57}H_{48}S_2]^+$).

Additional graphs and data

NMR Spectra

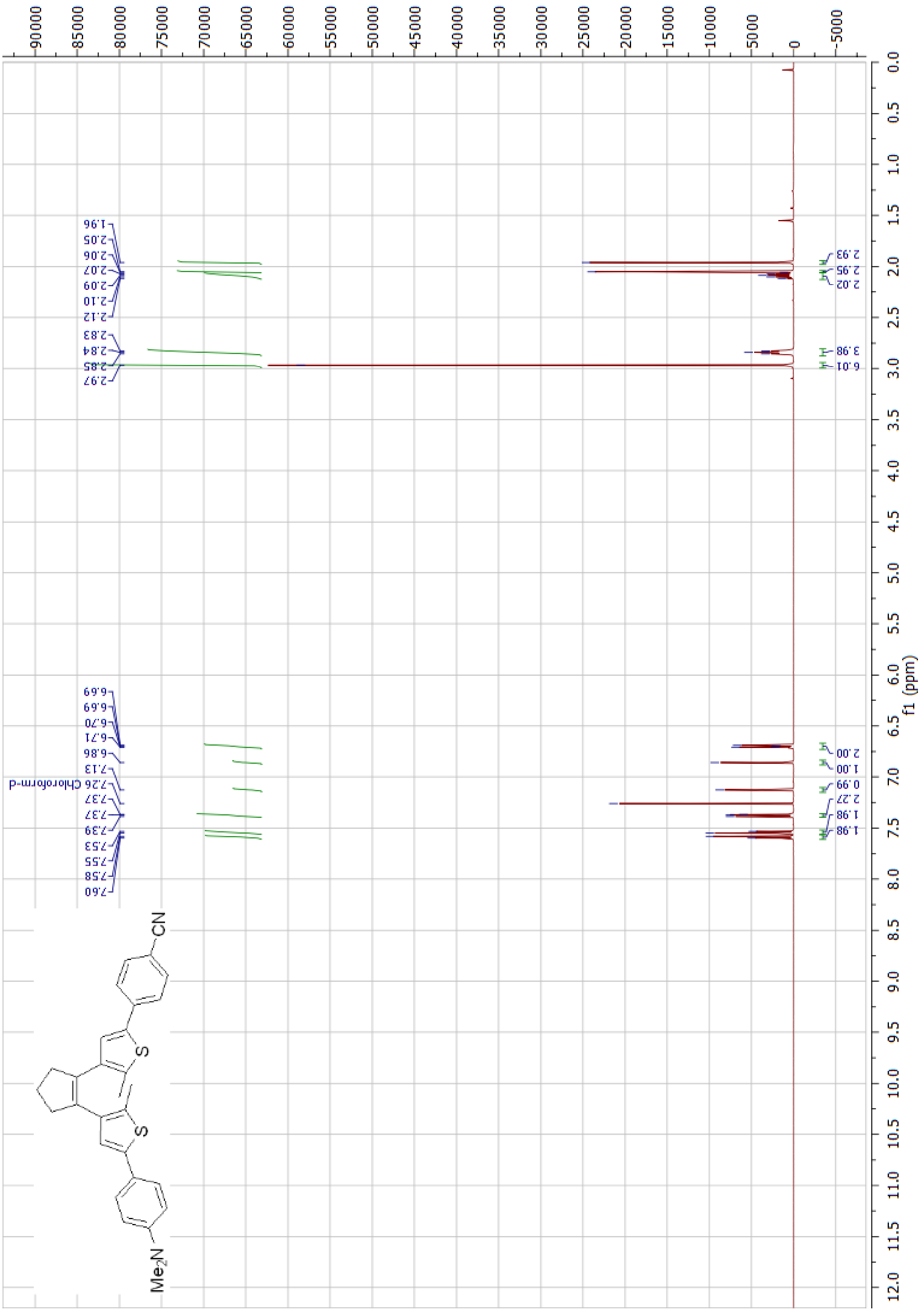

Figure S1: ^{1}H NMR of **Me_2NPh-DTE-PhCN** (500 MHz, $CDCl_3$).

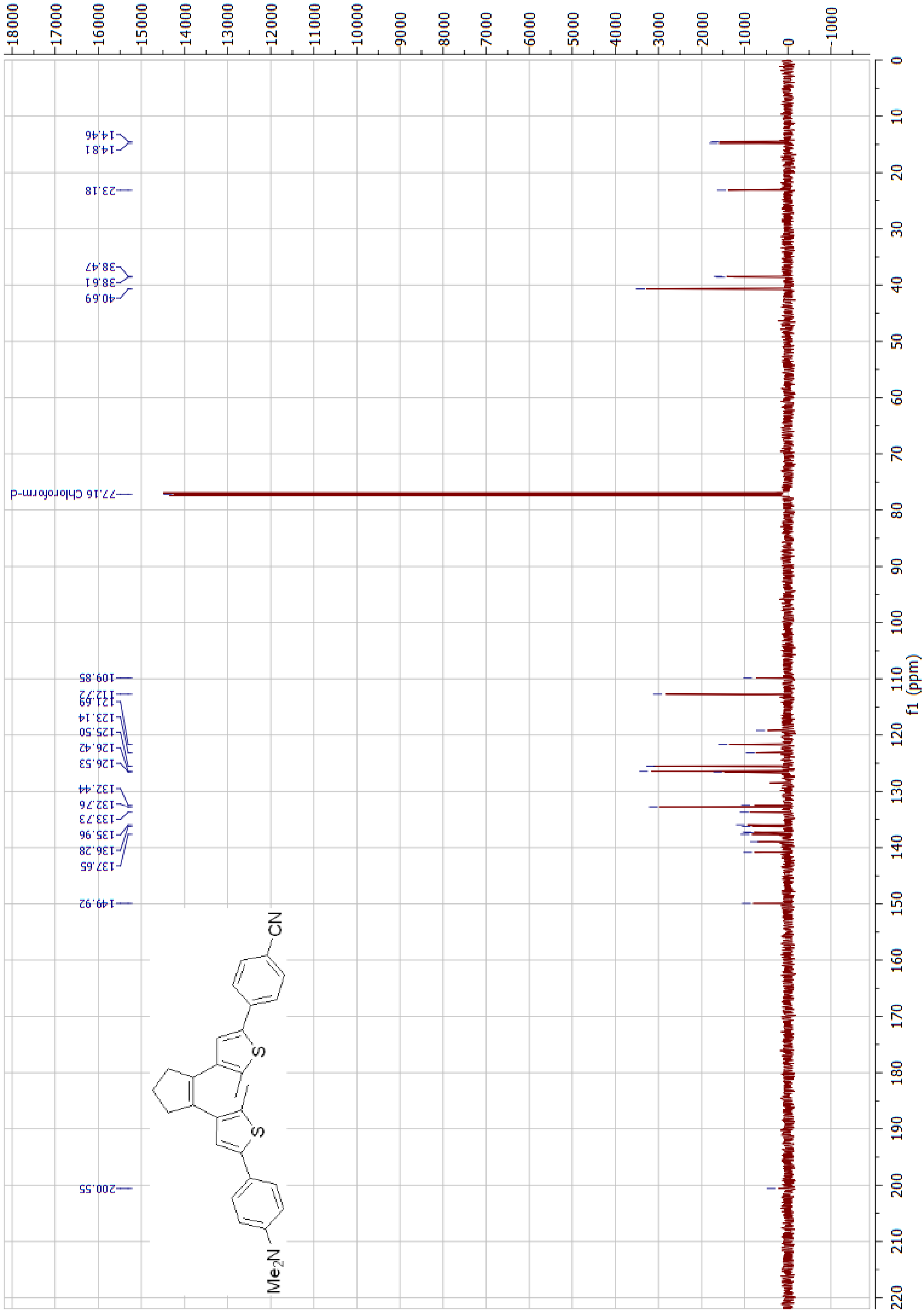

Figure S2: ^{13}C NMR of **Me$_2$NPh-DTE-PhCN** (126 MHz, CDCl$_3$).

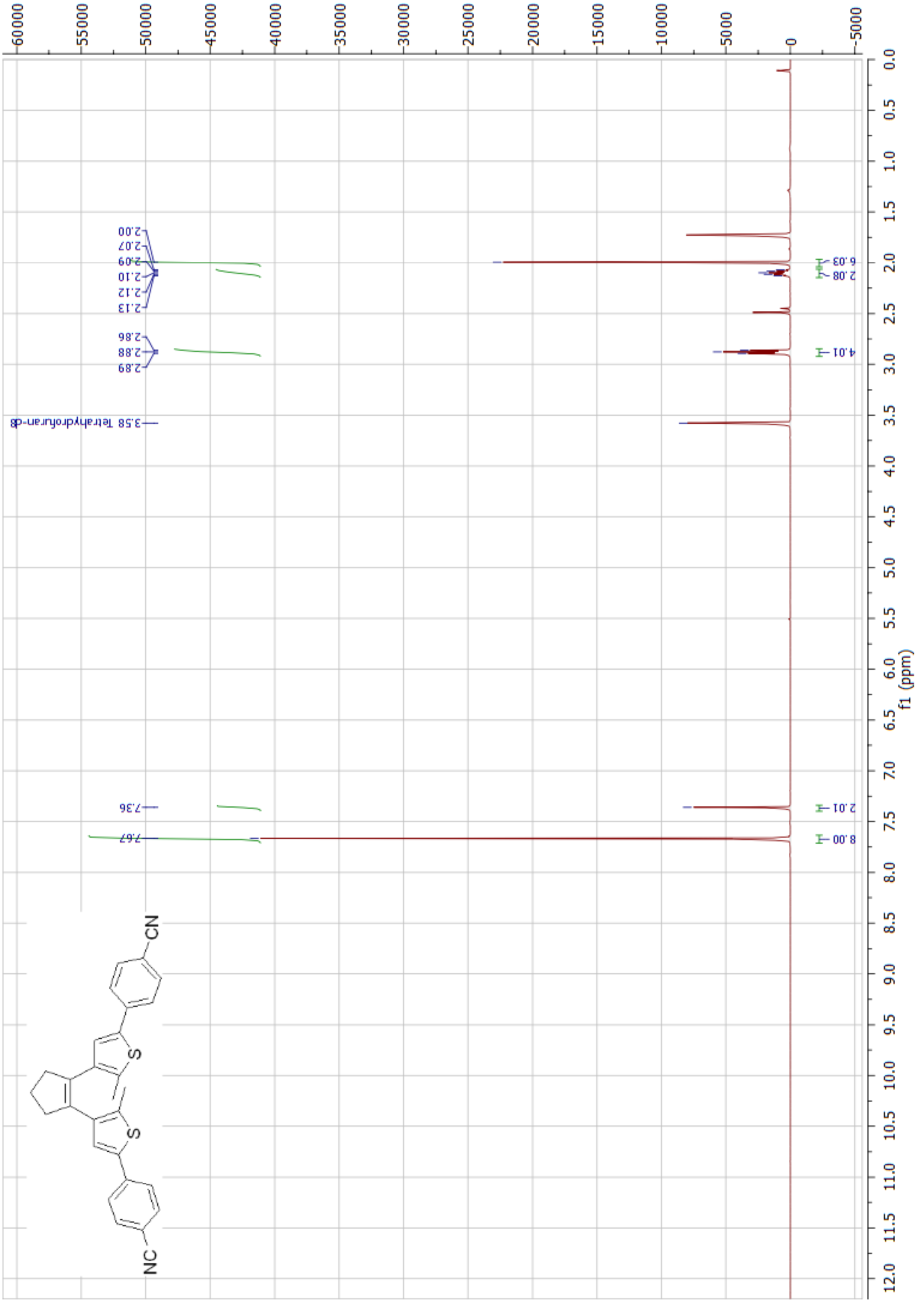

Figure S3: ^{1}H NMR of **DTE-PhCN** (500 MHz, THF-d_8).

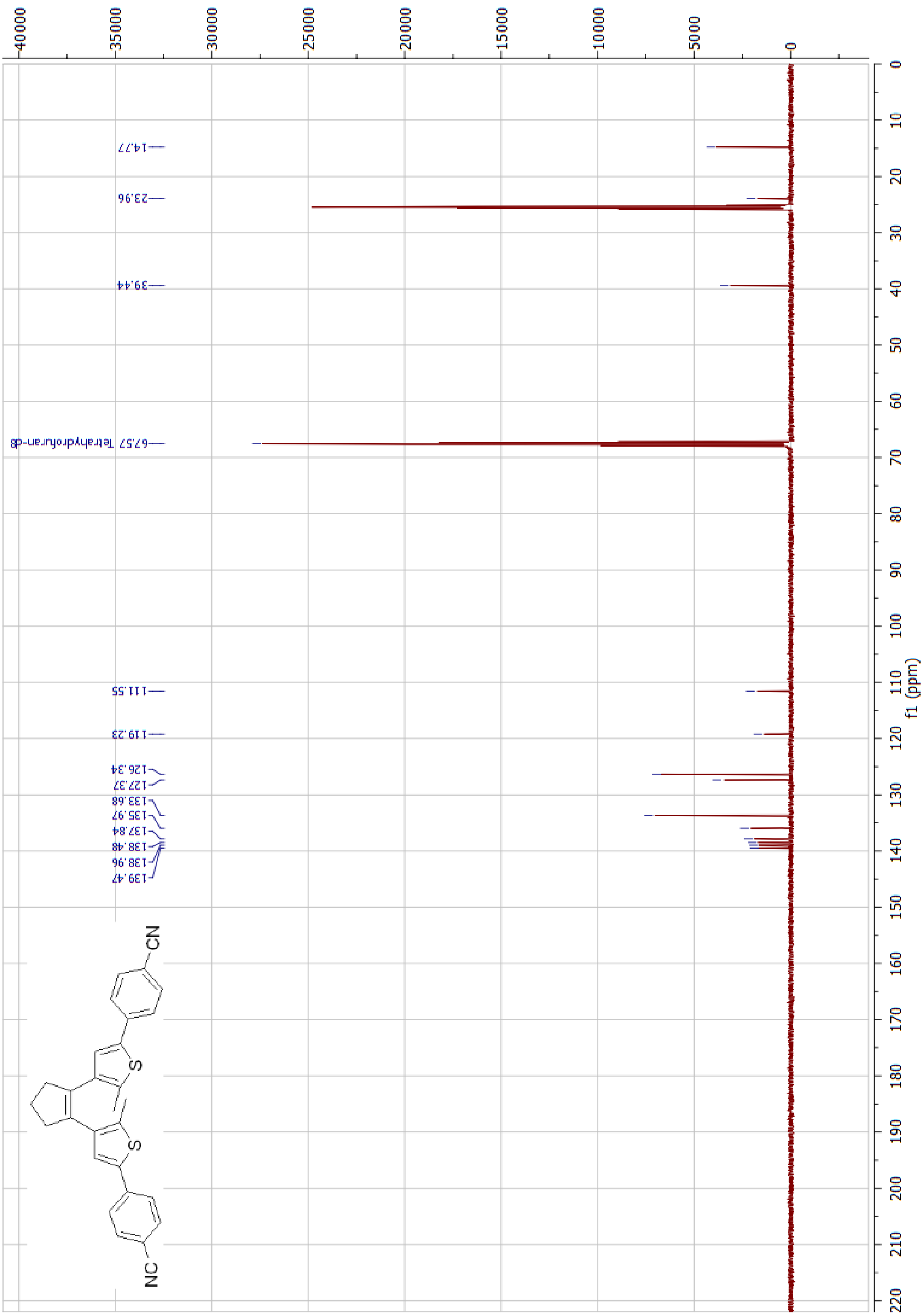

Figure S4: ^{13}C NMR of **DTE-PhCN** (126 MHz, THF-d_8).

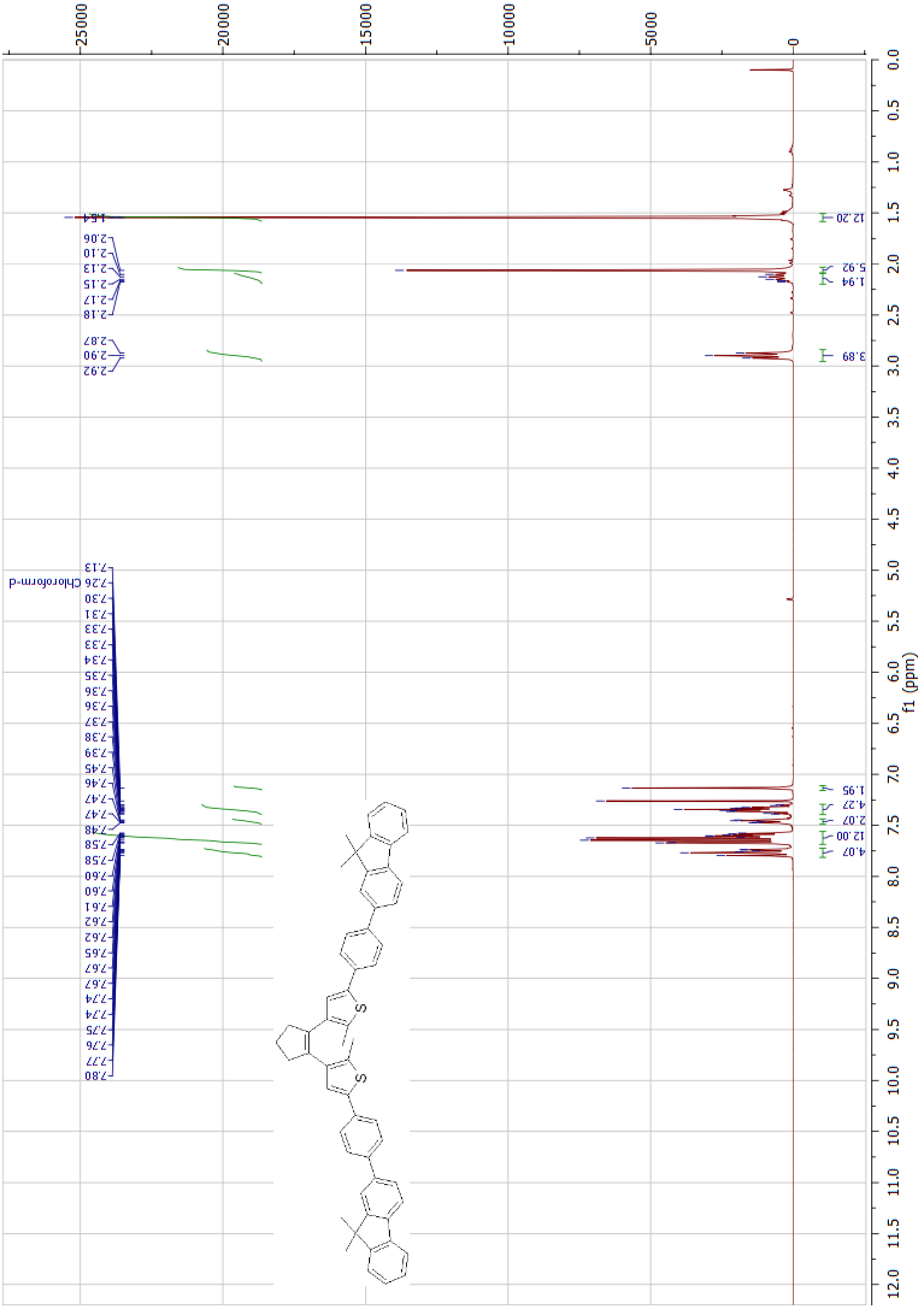

Figure S5: ^{1}H NMR of **DTE-PhFluorene** (300 MHz, $CDCl_3$).

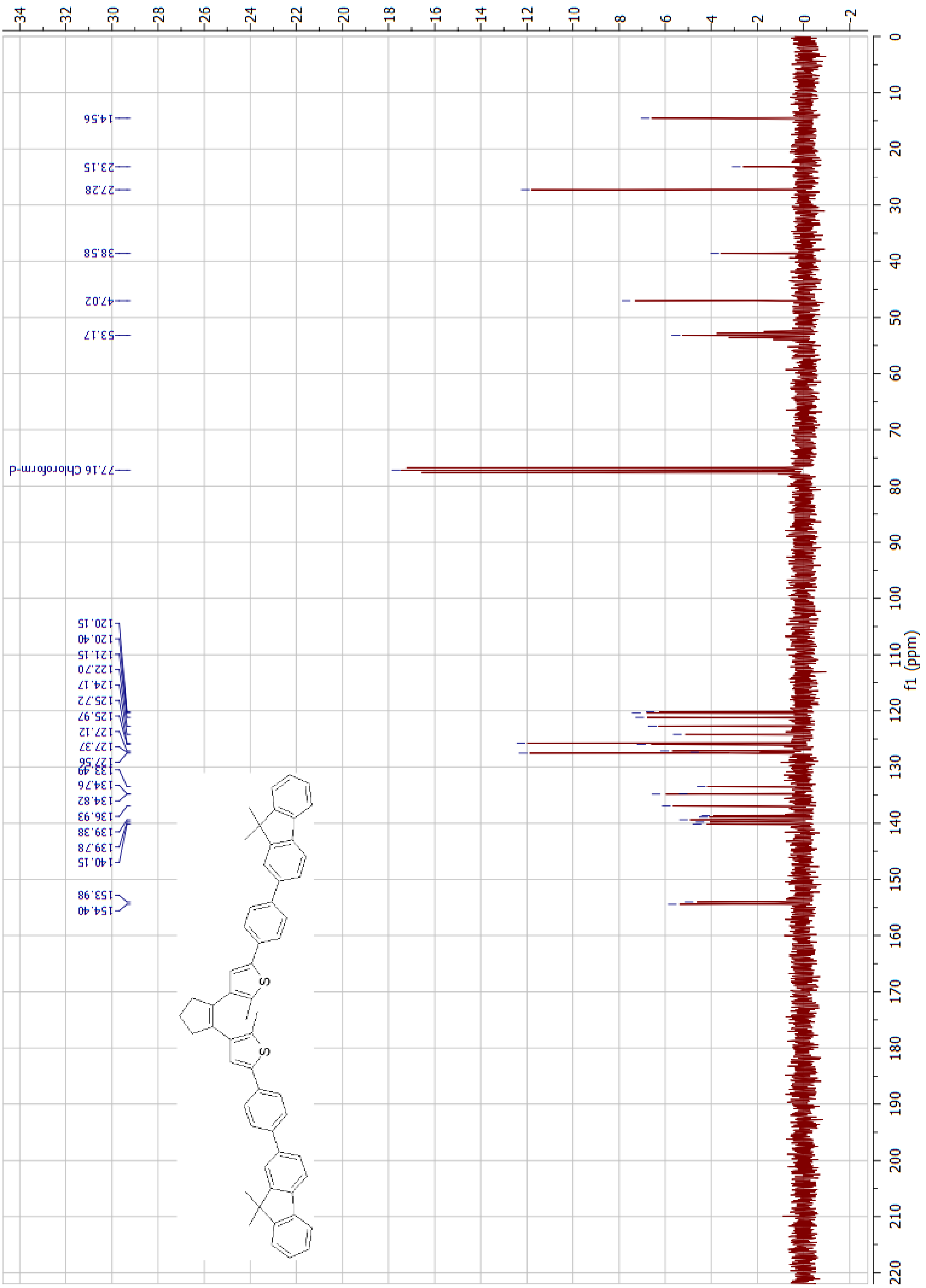

Figure S6: ^{13}C NMR of **DTE-PhFluorene** (75.5 MHz, $CDCl_3$).

UV–vis measurements

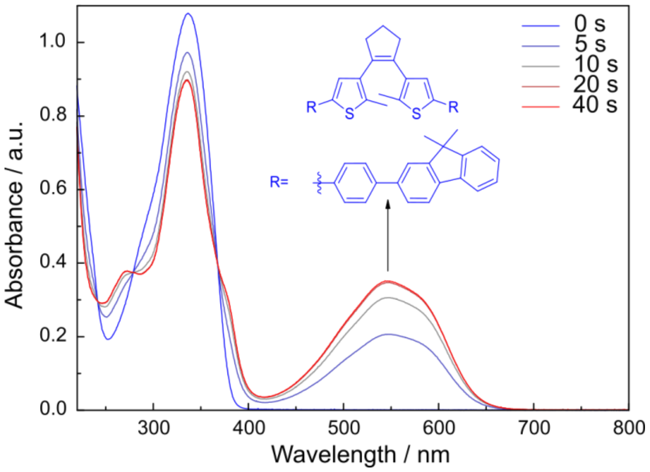

Figure S7: UV–vis spectra of ***O*-DTE-PhFluorene** in MeCN (c = 1.40·10^{-5} M, 25 °C), irradiated with UV light (1000 W Xe(Hg) lamp, 350 nm interference filter) until reaching the photostationary state (PSS).

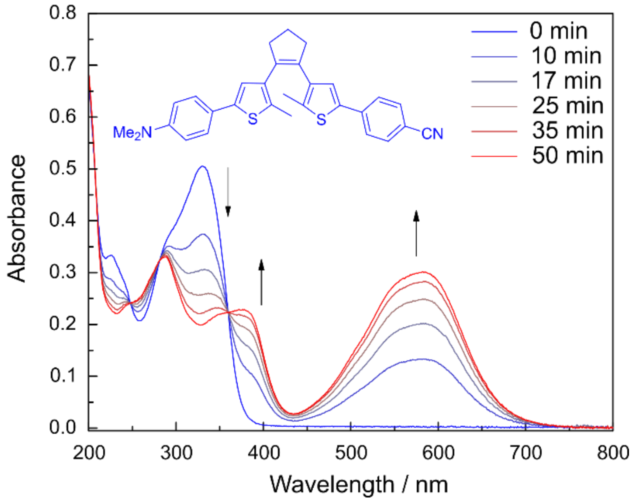

Figure S8: UV–vis spectra of ***O*-Me$_2$NPh-DTE-PhCN** in MeCN (c = 1.24·10^{-5} M, 25 °C) under irradiation with 313 nm light (500 W mercury lamp, monochromator) until reaching the PSS.

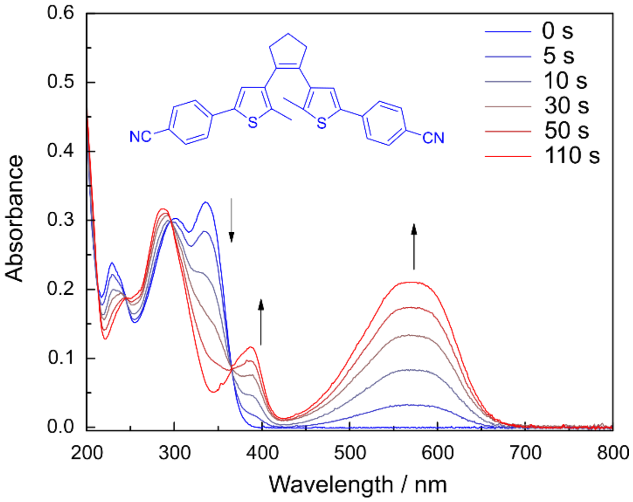

Figure S9: UV–vis spectra of ***O*-DTE-PhCN** in MeCN (c = 9.21·10^{-6} M, 25 °C) under irradiation with 313 nm light (500 W mercury lamp, monochromator) until reaching the PSS.

Redoxpotentials

Table S1: Peak potentials of sDTE and reference compounds vs Fc/Fc^{+}. Determined in MeCN with 0.1 M Bu_4NPF_6.

Compound	***E*** E_p^a [V]	***Z*** E_p^a [V]	***C*** E_p^{a1} [V]	***C*** E_p^{a2} [V]	***Z*** E_p^c [V]	***C*** E_p^c [V]	Cyclization conditions
**sDTE$_{55}$-Me**	0.567	-	-	-	-	-	none
**sDTE$_{66}$-Me**	0.517	0.494	-0.126	0.168	-	-	ox.
**sDTE(Me$_2$)$_{66}$-Me**	0.601	0.561	-0.055	0.292	< -2.8	-	ox.
**sDTE$_{77}$-Me**	0.885	0.675	-0.056	0.053	< -2.8	-	ox.
**sDTE$_{66}$-Ph(CF$_3$)$_2$**	-	0.785	0.152	0.462	< -2.8	-2.078	ox.
**sDTE$_{66}$-PhOMe**	-	0.455	-0.109	0.083	< -2.8	-2.534	ox.
**sDTE$_{66}$-PhBr**	-	0.607	0.055	0.336	< -2.8	-2.340	ox.
**sDTE$_{66}$-PhBr**[a)]	-	0.529	-0.045	0.352	< -2.5	-2.339	ox.
**sDTE$_{66}$-PhCN**	0.635	0.651	0.098	0.389	-2.526[b)]	-1.902[c)]	ox./red.
**sDTE$_{66}$-PhCN**[a)]	0.603	0.609	0.066	0.466	< - 2.5[d)]	-2.000	ox./red[d)]
**sDTE$_{77}$-Ph(CF$_3$)$_2$**	-	1.083	0.202	0.365	-	-2.079	ox.
**DTE-Me**	-	0.742	-0.012	0.248	< -2.8	-2.750	ox.
**DTE-PhCN**	-	0.930	0.180	0.444	-2.436	-1.816	ox./red.
**Me$_2$NPh-DTE-PhCN**	-	0.208[e)]	-0.145	0.075	-2.457	-1.977[f)]	ox.
**DTE-PhFluorene**[a)]	-	0.717	0.006	0.199	-2.317[g)]	-2.072[g)]	ox./red.
**Ethene-Me**	0.590	0.682	-	-	< -2.8	-	none
**Butene-Me**	0.891	0.731	0.030	0.231	< -2.8	-	(ox.)

[a)] in DCM [b)] E_p^c (***E***): -2.510 V[d)]; -2.522 V (in DMF) [c)] E_p^c (***C***): -1.904 V (in DMF) [d)] reduction of compound and electrolyte occur at similar potentials [e)] E_p^{a2} (***Z***): 0.624 V [f)] E_p^{c2} (***C***): -2.234 V [g)] in DMF

CV measurements

All cyclovoltammograms are measured in MeCN, if not mentioned otherwise.

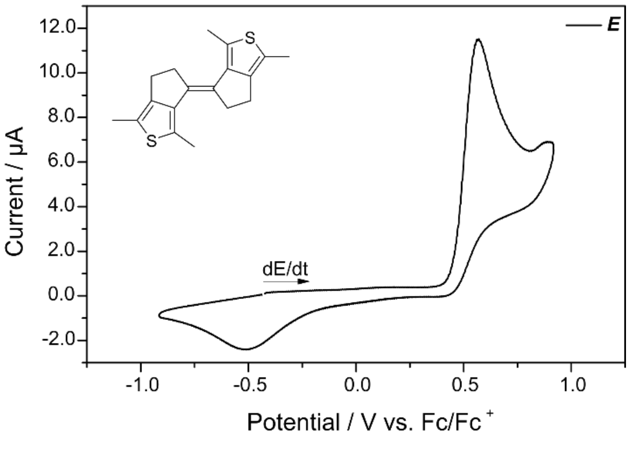

Figure S10: Cyclic voltammogram of ***E*-sDTE$_{55}$-Me** (c = 1·10^{-3} M).

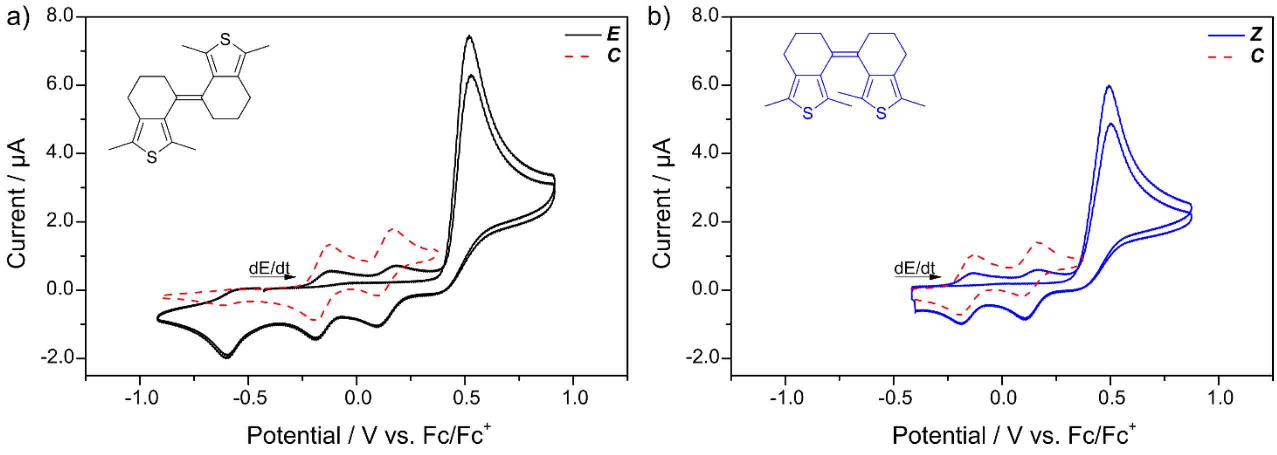

Figure S11: Two consecutive cyclovoltammetric cycles of ***E*-sDTE$_{66}$-Me** (c = 1·10^{-3} M) and ***Z*-sDTE$_{66}$-Me** (c = 7.5·10^{-4} M).

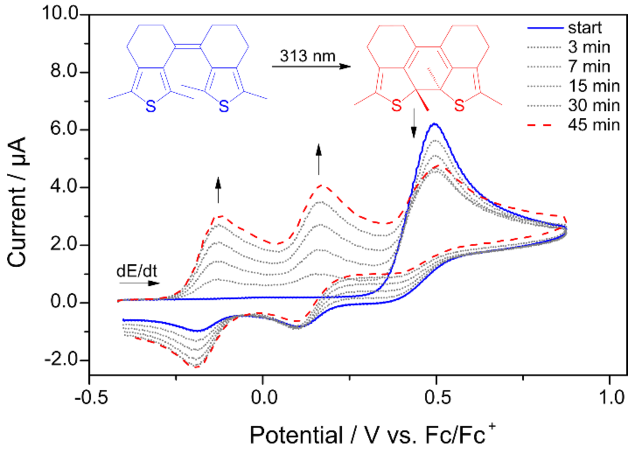

Figure S12: Changes in the cyclic voltammogram during cyclization of ***Z*-sDTE$_{66}$-Me** (c = 1·10^{-3} M in DCM) by irradiation with 313 nm light.

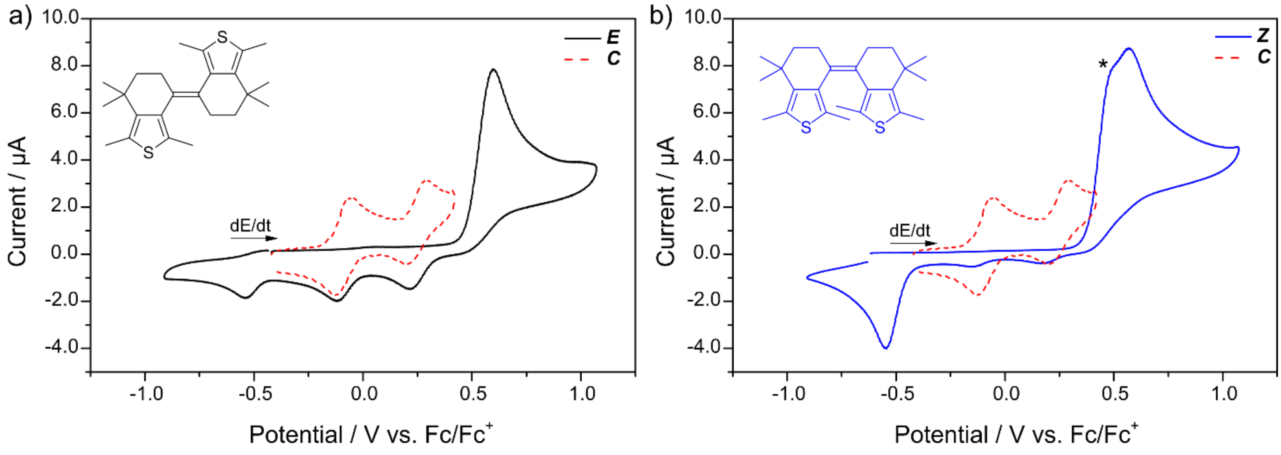

Figure S13: Cyclic voltammogram of ***E*-sDTE(Me$_2$)$_{66}$-Me** and ***Z*-sDTE(Me$_2$)$_{66}$-Me** (c = 1·10^{-3} M). As it is not obtained in synthesis, ***Z*-sDTE(Me$_2$)$_{66}$-Me** was generated in situ by a cyclization/cycloreversion sequence starting from ***E*-sDTE(Me$_2$)$_{66}$-Me**. The shoulder (*) is attributed to byproducts formed during irradiation.

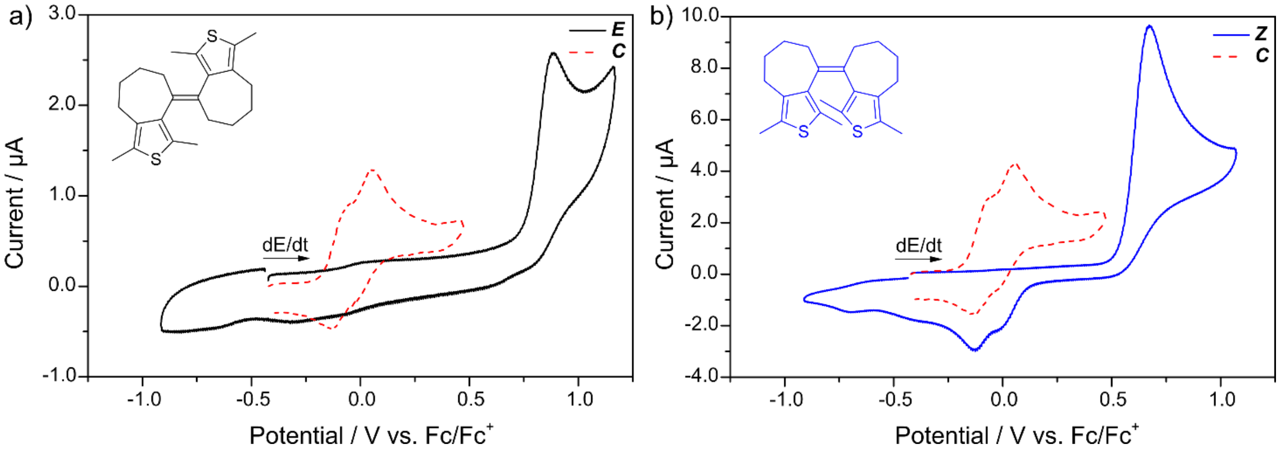

Figure S14: Cyclic voltammogram of ***E*-sDTE$_{77}$-Me** (c = 5·10^{-4} M) and ***Z*-sDTE$_{77}$-Me** (c = 1·10^{-3} M).

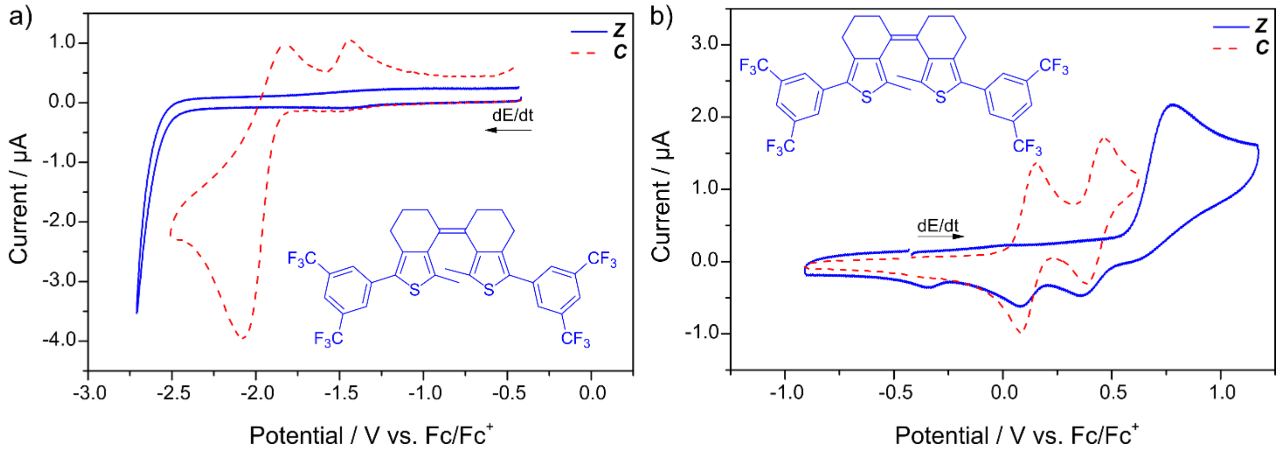

Figure S15: Cyclic voltammogram of ***Z*-sDTE$_{66}$-Ph(CF$_3$)$_2$** (c = 5·10^{-4} M).

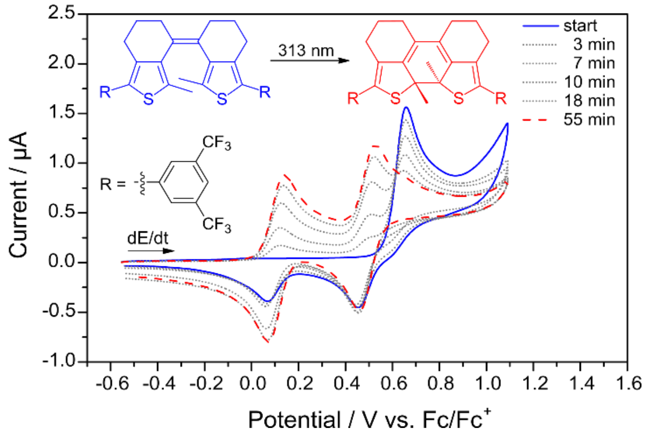

Figure S16: Changes in the cyclic voltammogram during cyclization of ***Z*-sDTE$_{66}$-Ph(CF$_3$)$_2$** (c = 1·10^{-3} M in DCM) by irradiation with 313 nm light.

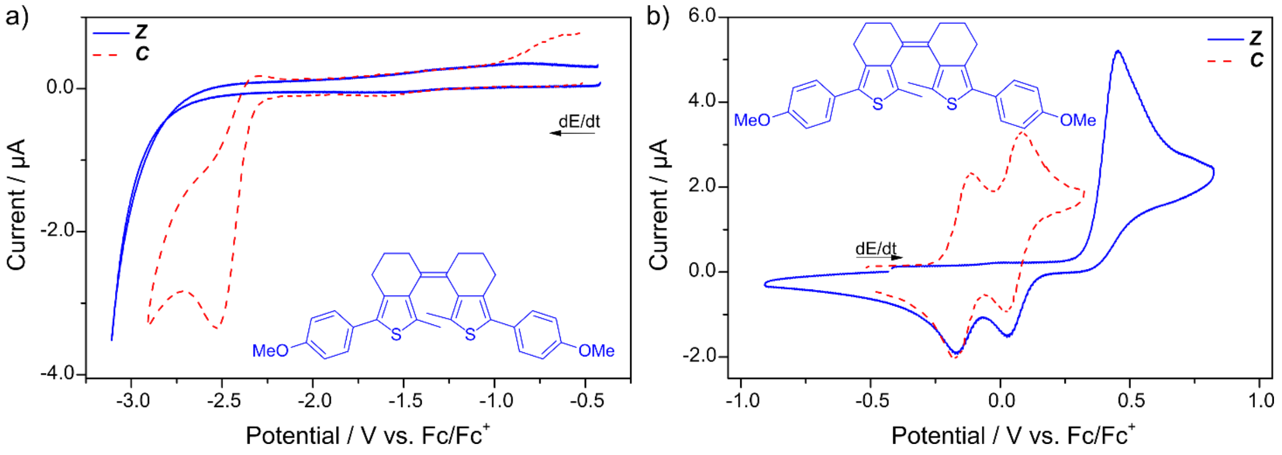

Figure S17: Cyclic voltammogram of ***Z*-sDTE$_{66}$-PhOMe** (c = 1·10^{-3} M).

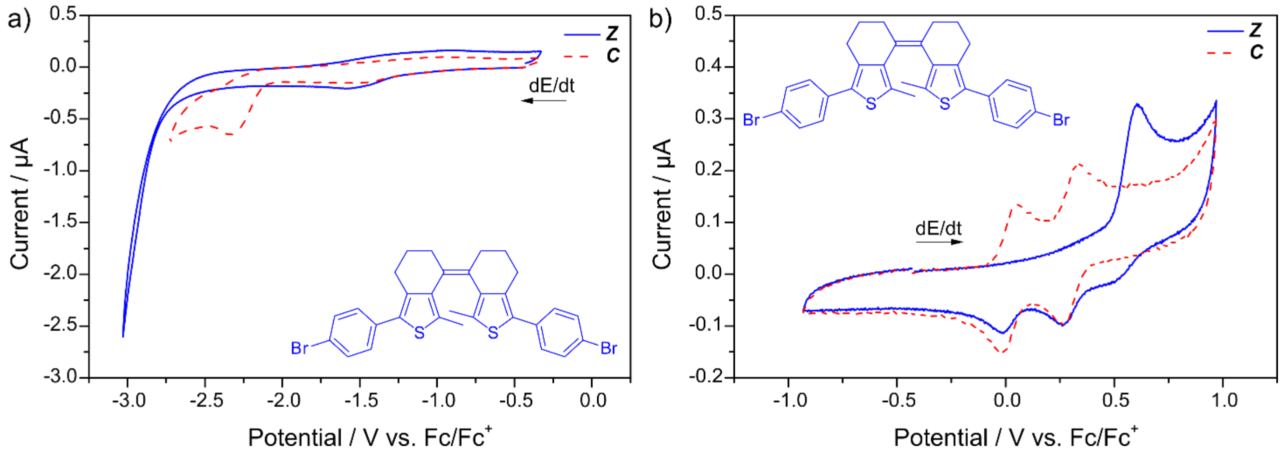

Figure S18: Cyclic voltammogram of ***Z*-sDTE$_{66}$-PhBr** (c < 5·10^{-4} M). Voltammogram obtained from DCM solution is similar.

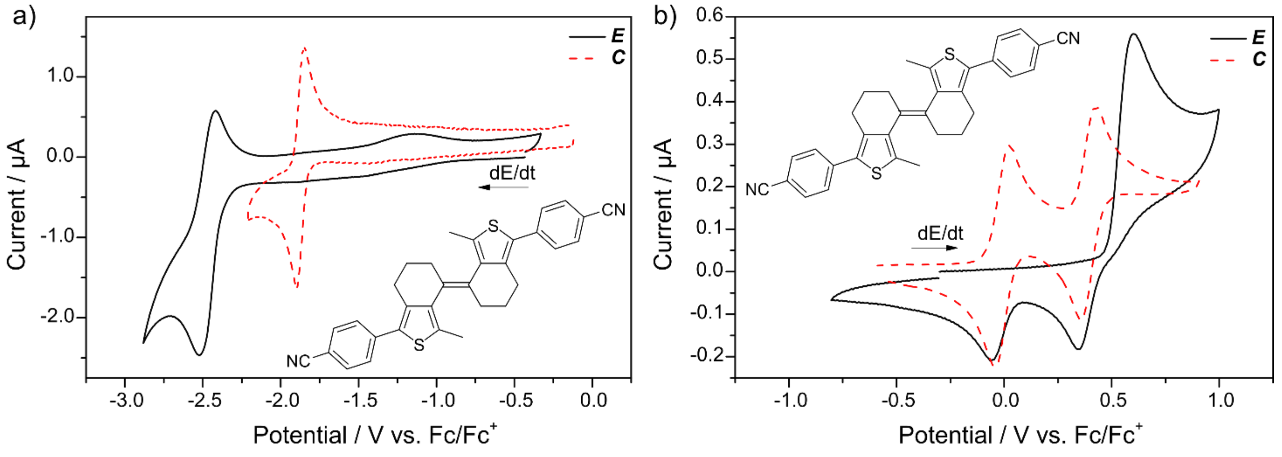

Figure S19: Cyclic voltammogram of ***E*-sDTE$_{66}$-PhCN** (c = 5·10^{-4} M) a) in DMF and b) in DCM. Measurements in a Microcell [4] (rhd-instruments; Pt-disc d = 0.25 mm, scan rate dE/dt = 1 V s^{-1}).

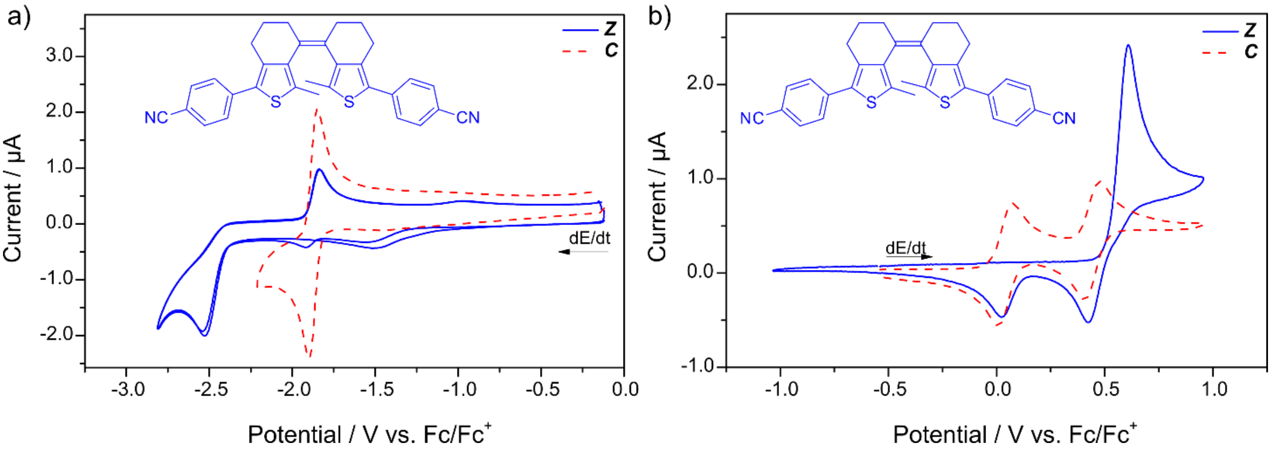

Figure S20: Cyclic voltammogram of ***Z*-sDTE$_{66}$-PhCN** (c = 1·10^{-3} M) a) in MeCN and b) in DCM.

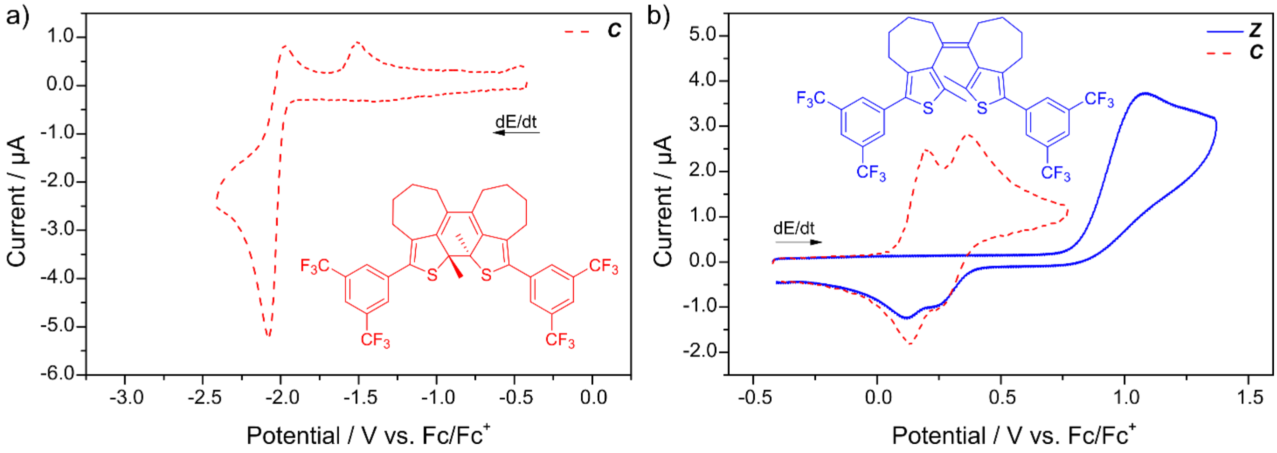

Figure S21: Cyclic voltammogram of ***Z*-sDTE$_{77}$-Ph(CF$_3$)$_2$** (c = 5·10^{-4} M).

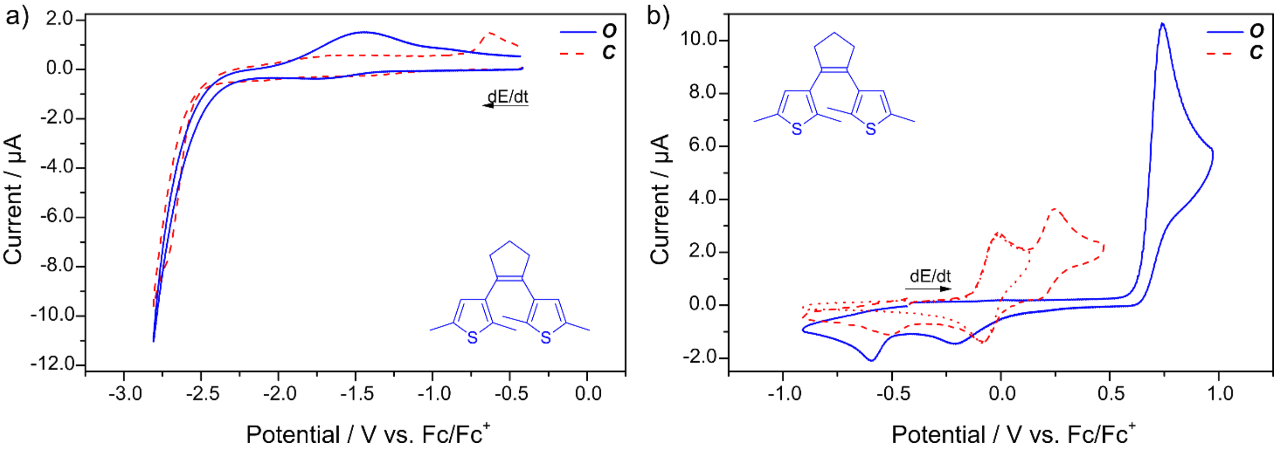

Figure S22: Cyclic voltammogram of ***O*-DTE-Me** (c = 1·10^{-3} M).

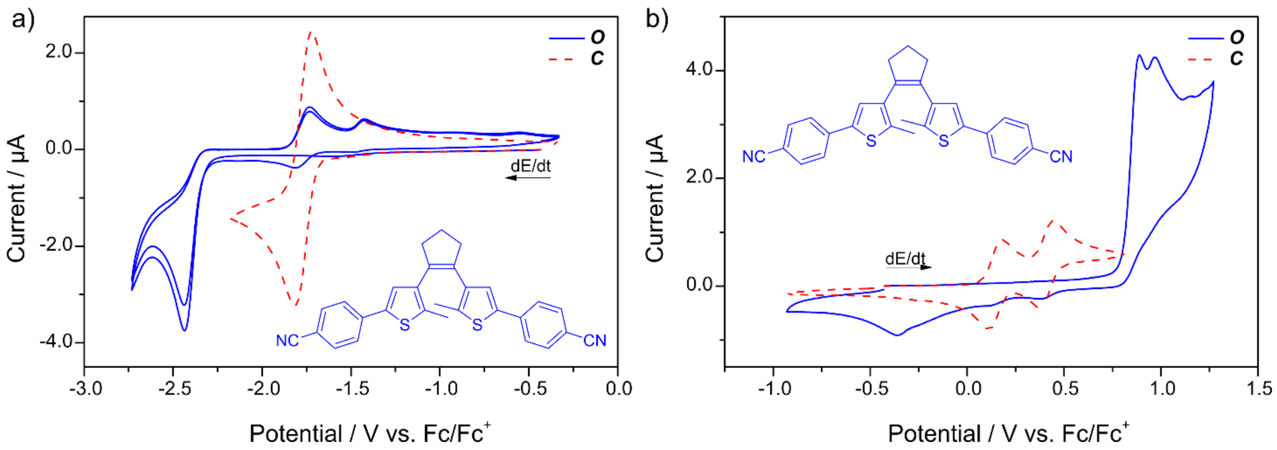

Figure S23: Two consecutive cyclovoltammetric cycles and cyclic voltammogram of ***O*-DTE-PhCN** (c = 1·10^{-3} M).

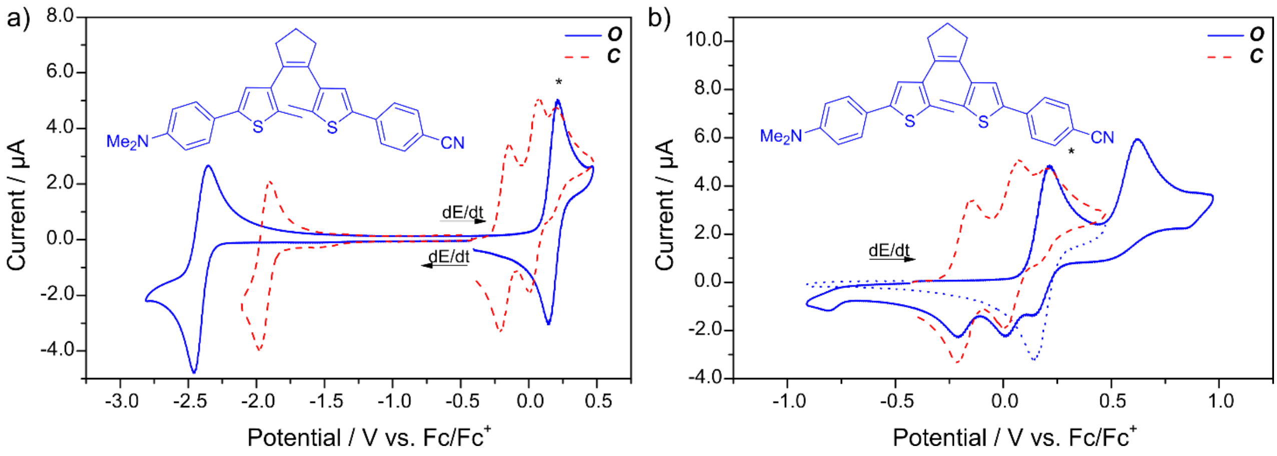

Figure S24: Cyclic voltammogram of ***O*-Me$_2$NPh-DTE-PhCN** (c = 1·10^{-3} M). The first oxidation and reduction of ***O*-Me$_2$NPh-DTE-PhCN** is reversible. The shoulder (*) in the cyclovoltammograms of ***C*** is attributed to residual open isomer.

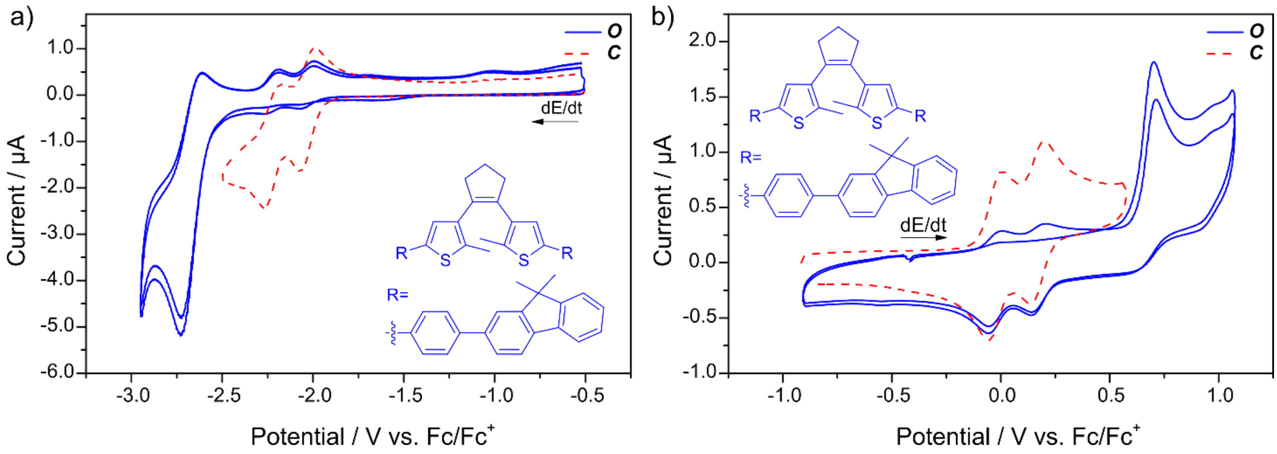

Figure S25: Two consecutive cyclovoltammetric cycles of ***O*-DTE-PhFluorene** a) in DMF (c = 1·10^{-3} M) and b) in DCM (c = 7·10^{-4} M).

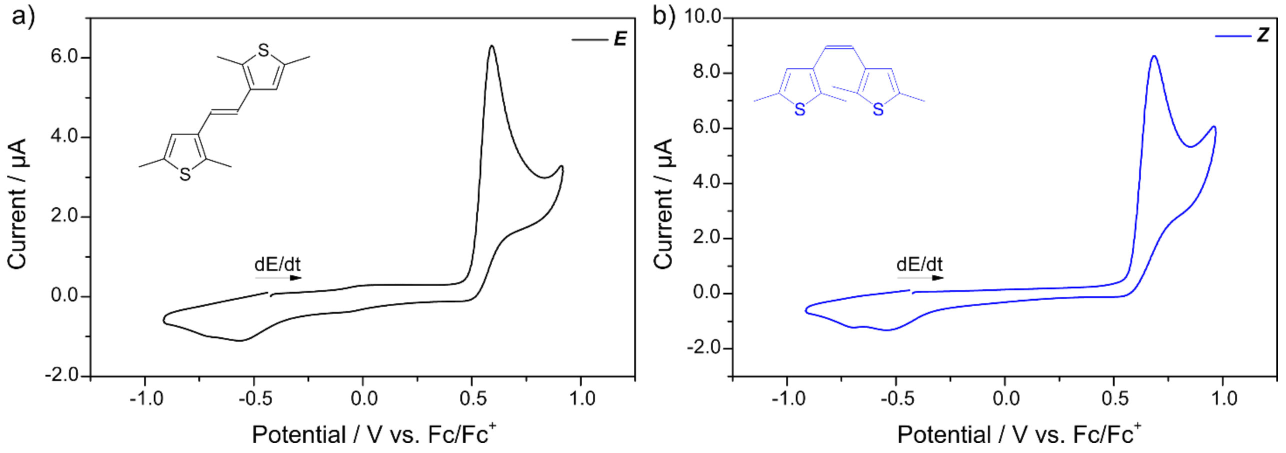

Figure S26: Cyclic voltammogram of ***E*-ethene-Me** and ***Z*-ethene-Me** ($c = 1{\cdot}10^{-3}$ M).

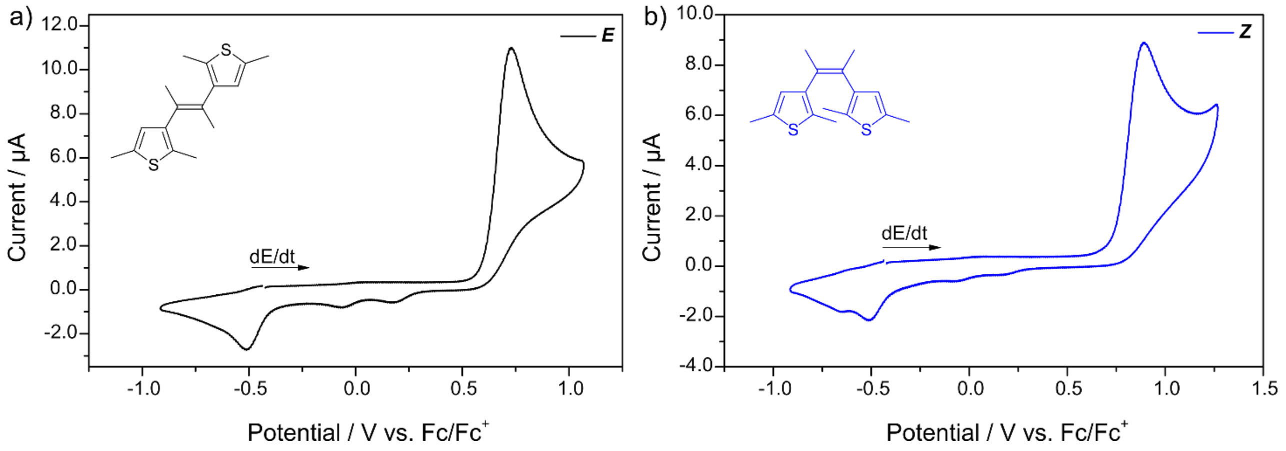

Figure S27: Cyclic voltammogram ***E*-butene-Me** and ***Z*-butene-Me** ($c = 1{\cdot}10^{-3}$ M).

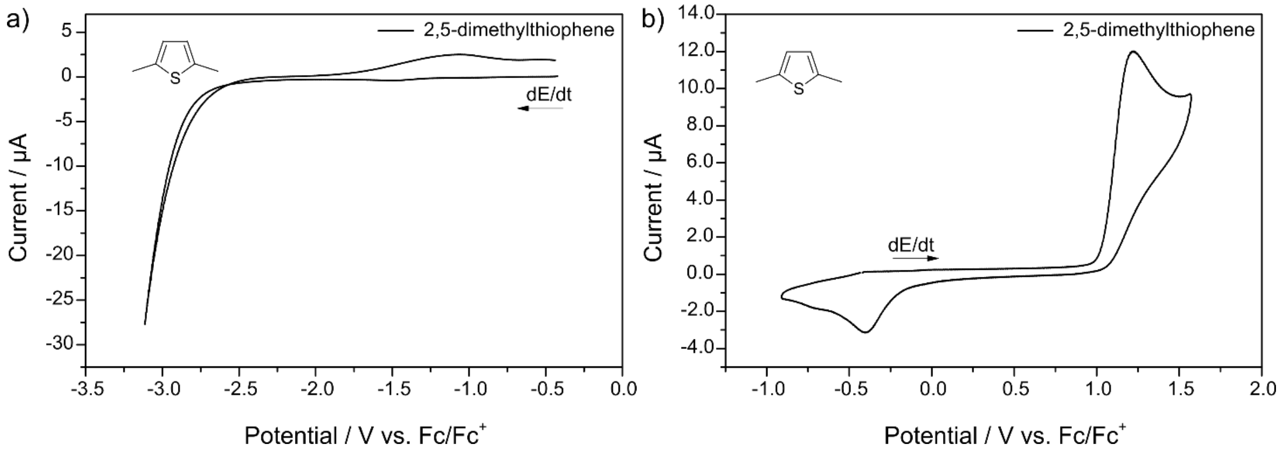

Figure S28: Cyclic voltammogram of 2,5-dimethylthiophene ($c = 1{\cdot}10^{-3}$ M).

SEC Measurements

All SEC measurements are carried out in MeCN, if not mentioned otherwise.

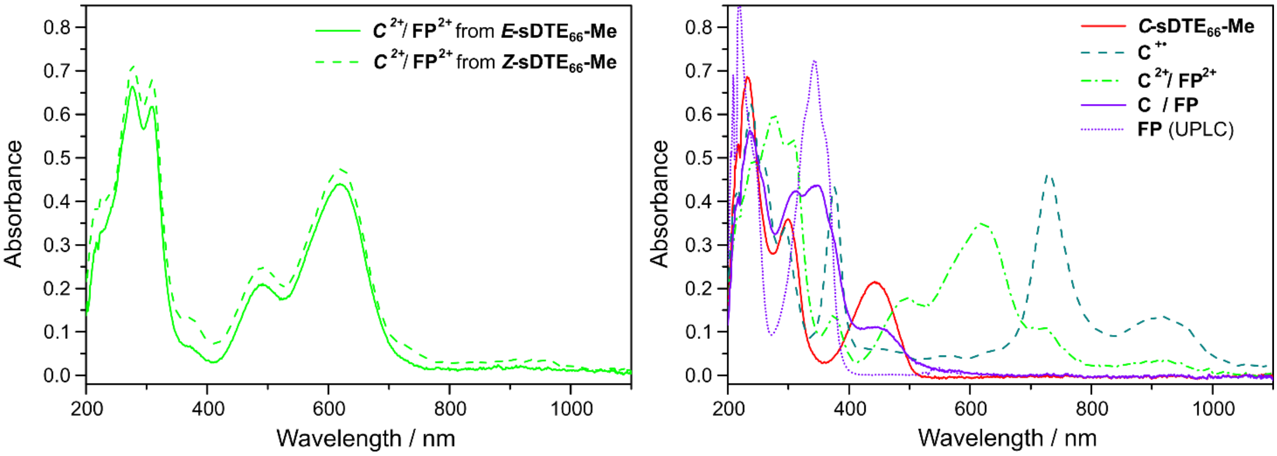

Figure S29: SEC of **sDTE$_{66}$-Me.** a (left)) Comparison of UV–vis spectra of ***C***$^{2+}$/**FP**$^{2+}$ formed from ***E***- and ***Z*-sDTE$_{66}$-Me**. b (right)) Spectra of all species involved at the respective redox wave maximum. For comparison, the absorption spectrum of **FP** extracted from the UPLC (Figure S41e, UPLC 2) is added as reference. The interaction of eluent mixture (MeCN/H_2O/formic acid) with **FP**$^{2+}$ solution injected accounts for the same transformation as under reductive conditions.

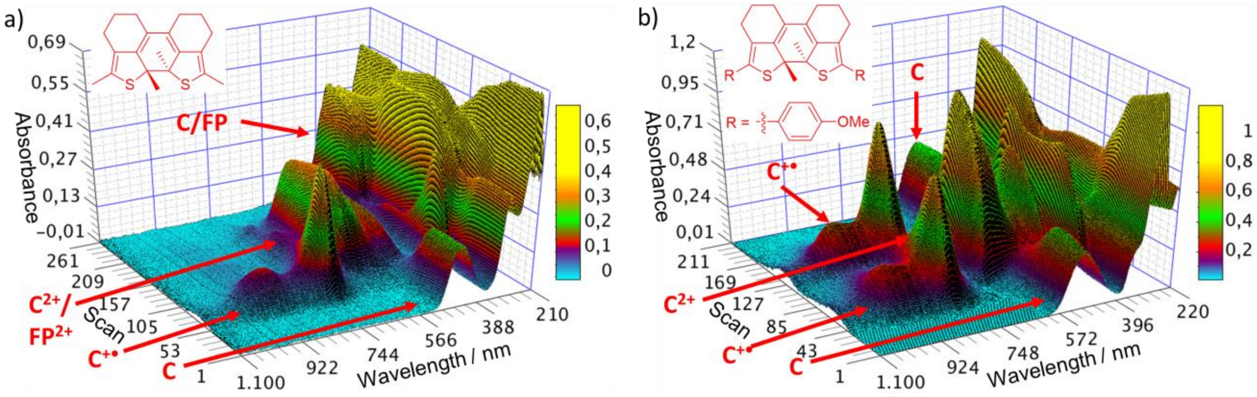

Figure S30: Full SEC cycles. a) The oxidation of ***C*-sDTE$_{66}$-Me** (c = 5·10^{-4} M) is not reversible and leads to formation of a mixture of ***C*** and **FP**. Start and return potentials at −0.52 V, 0.32 V and −0.73 V. b) For ***C*-sDTE$_{66}$-PhOMe** (c = 5·10^{-4} M) the formation of the hypsochromic cationic radical ***C***$^{+\bullet}$ and dicationic ***C***$^{2+}$ is fully reversible. Start and return potentials at −0.66 V, 0.34 V and −0.97 V.

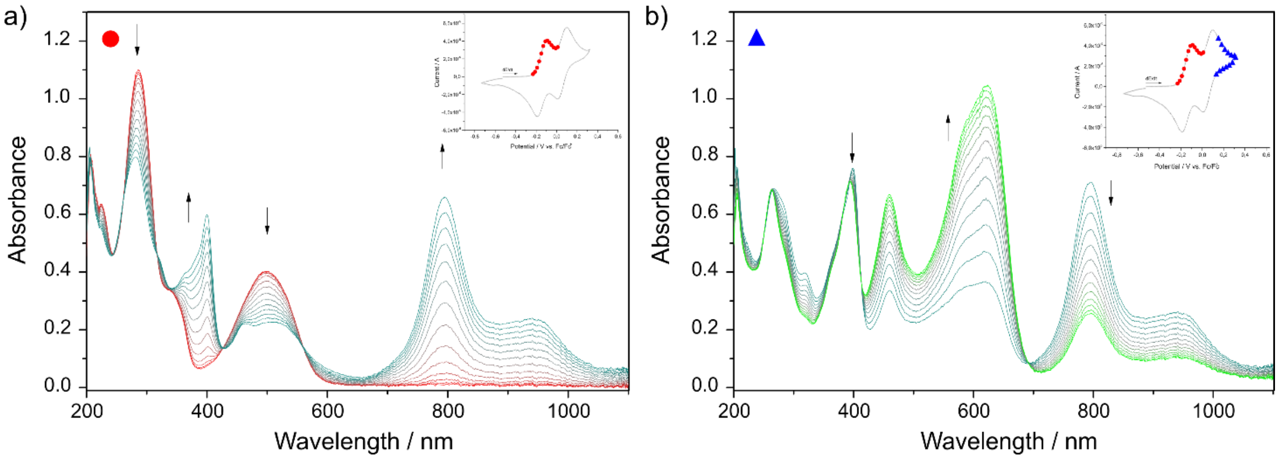

Figure S31: Changes in the UV–vis spectra during cyclic voltammetry of ***C*-sDTE$_{66}$-PhOMe** (c = 5.0·10^{-4} M). a) Build-up of the radical cation $\boldsymbol{C^{+\bullet}}$ and b) the dication $\boldsymbol{C^{2+}}$.

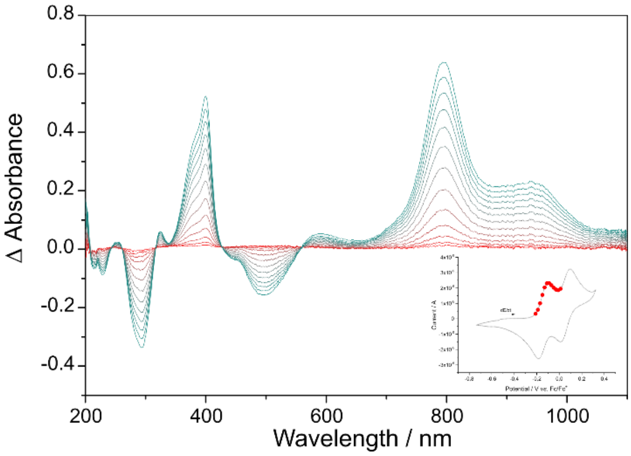

Figure S32: Measured difference spectra for the build-up of the radical cation $\boldsymbol{C^{+\bullet}}$ from ***C*-sDTE$_{66}$-PhOMe** (c = 5.0·10^{-4} M).

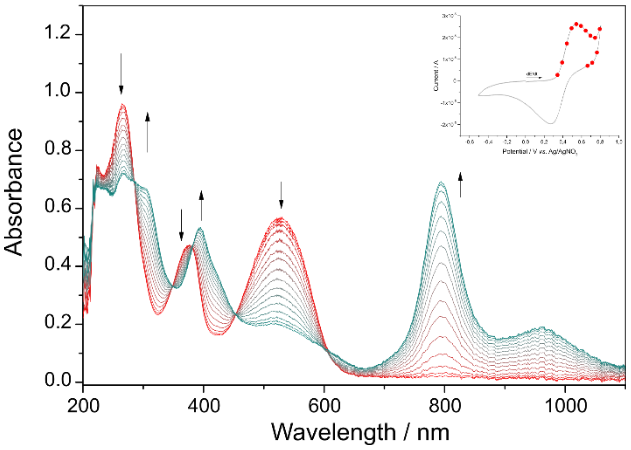

Figure S33: Changes in the UV–vis-spectra during cyclic voltammetry of ***C*-sDTE$_{66}$-Ph(CF$_3$)$_2$** (c = 5·10^{-4} M in DCM) for the build-up of the radical cation $\boldsymbol{C^{+\bullet}}$.

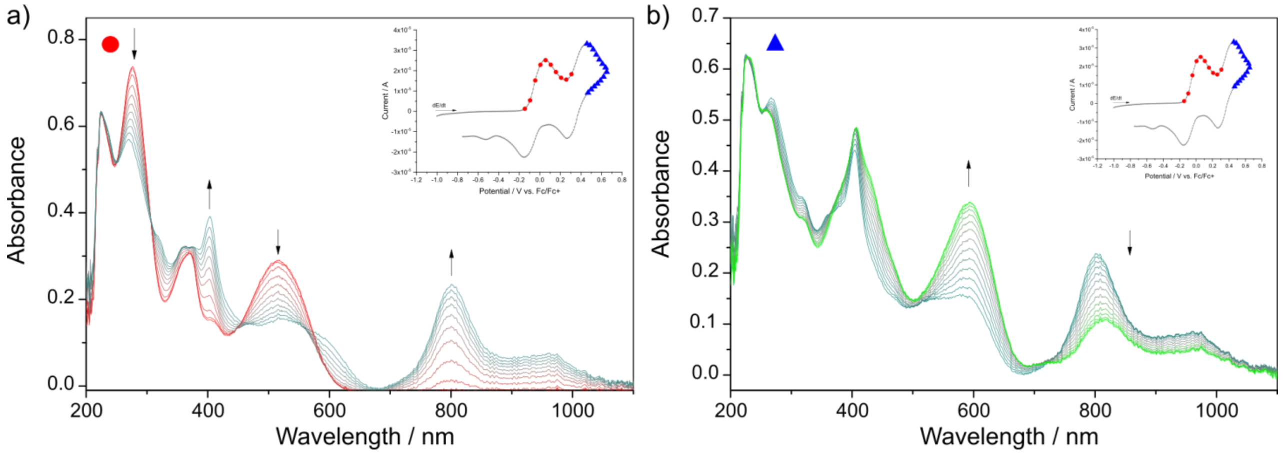

Figure S34: Changes in the UV–vis spectra during cyclic voltammetry of ***C*-sDTE$_{66}$-PhBr** (c = 5·10^{-4} M in DCM). a) Build-up of the radical cation $\boldsymbol{C^{+\bullet}}$ and b) the dication $\boldsymbol{C^{2+}}$.

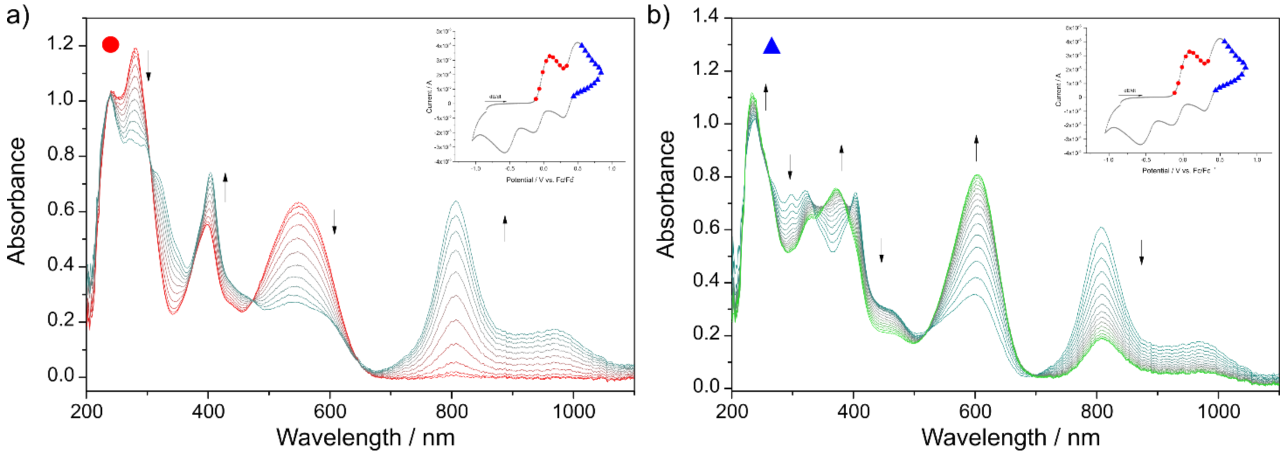

Figure S35: Changes in the UV–vis-spectra during cyclic voltammetry of ***C*-sDTE$_{66}$-PhCN** (c = 5·10^{-4} M in DCM). a) Build-up of the radical cation $\boldsymbol{C^{+\bullet}}$ and b) the dication $\boldsymbol{C^{2+}}$.

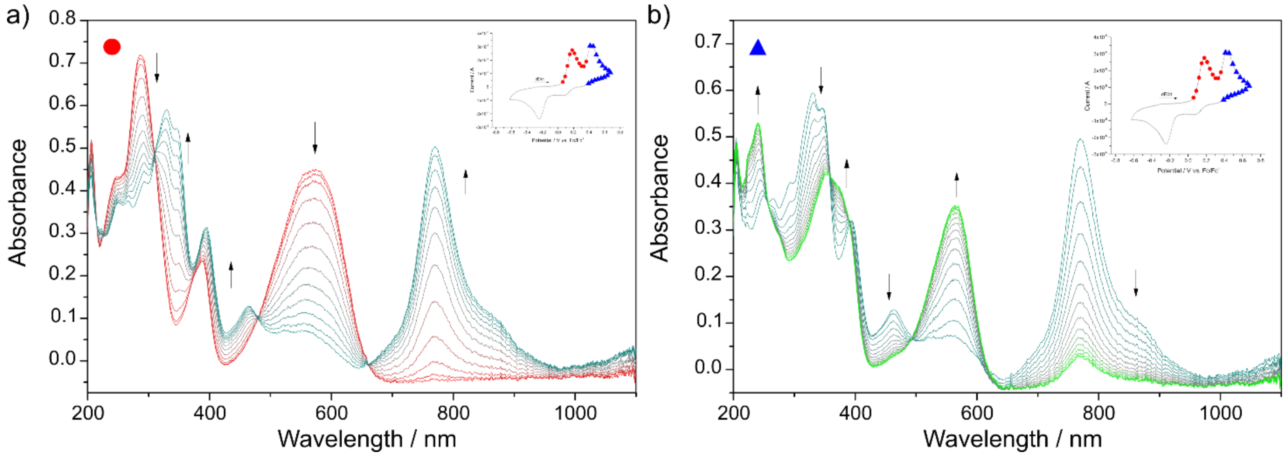

Figure S36: Changes in the UV–vis-spectra during cyclic voltammetry of ***C*-DTE-PhCN** (c = 5·10^{-4} M). a) Build-up of the radical cation $\boldsymbol{C^{+\bullet}}$ and b) the dication $\boldsymbol{C^{2+}}$.

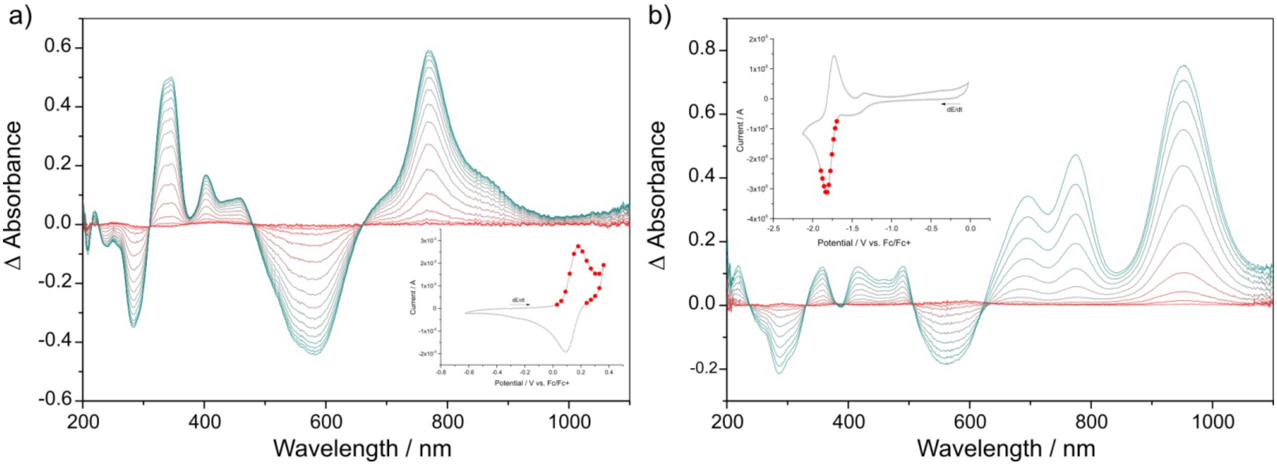

Figure S37: Measured difference spectra for a) the build-up of the radical cation $\boldsymbol{C^{+\bullet}}$ from ***C*-DTE-PhCN** (c = 5·10^{-4} M) and b) the radical anion $\boldsymbol{C^{-\bullet}}$.

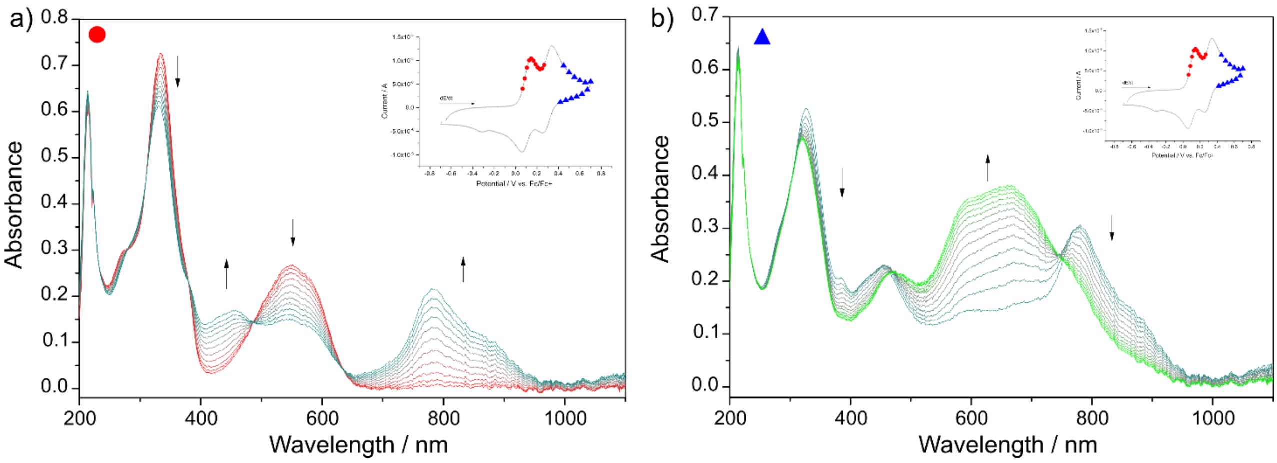

Figure S38: Changes in the UV–vis-spectra during cyclic voltammetry of ***C*-DTE-PhFluorene** (c = 2.1·10^{-4} M). a) Build-up of the radical cation $\boldsymbol{C^{+\bullet}}$ and b) the dication $\boldsymbol{C^{2+}}$.

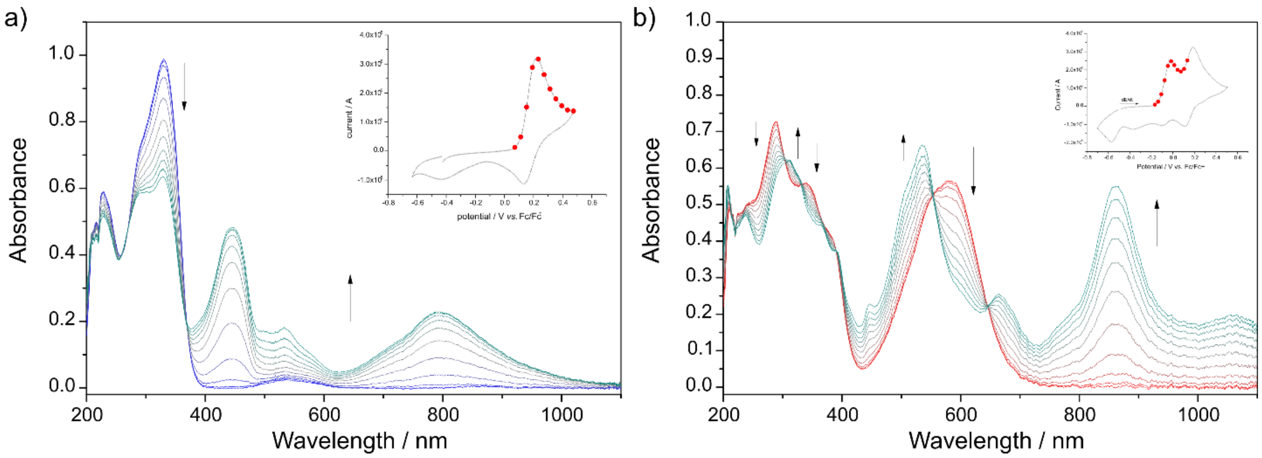

Figure S39: Changes in the UV–vis-spectra during cyclic voltammetry. a) Build-up of the radical cation $\boldsymbol{O^{+\bullet}}$ from ***O*-Me$_2$NPh-DTE-PhCN** (c = 6.8·10^{-4} M) and b) the radical cation $\boldsymbol{C^{+\bullet}}$ from ***C*-Me$_2$NPh-DTE-PhCN** (c = 3.8·10^{-4} M).

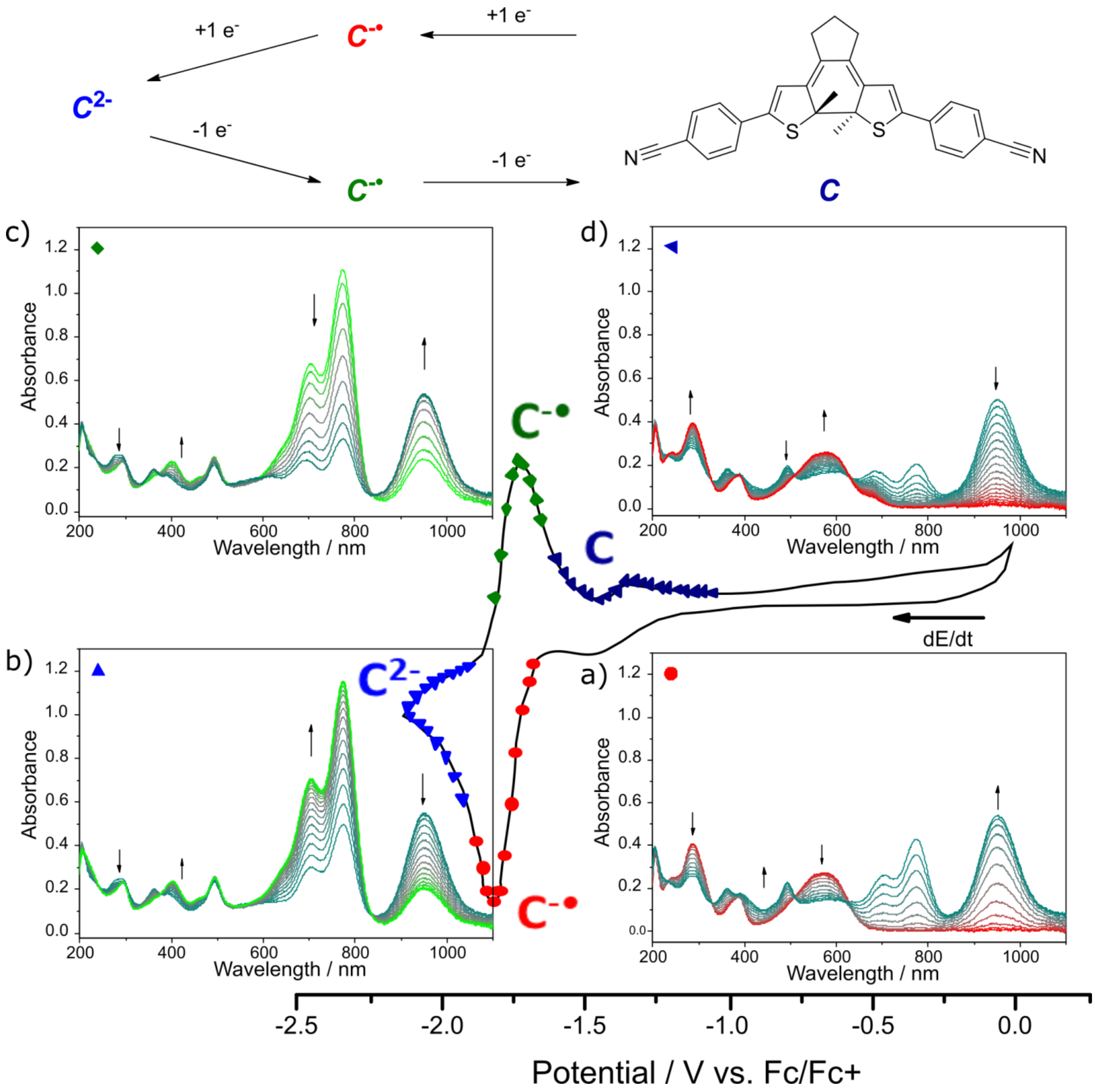

Figure S40: SEC analysis of ***C*-DTE-PhCN**. a) The initial formation of $\boldsymbol{C^{-\bullet}}$ is indicated by a continuous increase in the far red-shifted band at 953 nm, typical for radicals. It is accompanied by formation of $\boldsymbol{C^{2-}}$, as visible by its two absorption bands at 774 nm and 705 nm. b) Though not quantitively, $\boldsymbol{C^{-\bullet}}$ subsequently is depleted in favor of $\boldsymbol{C^{2-}}$, proving the parallel existence of both species. c) Re-oxidation stimulates the inverse shift of the equilibrium, d) with the offset of the oxidation wave showing the spectral fingerprint of the neutral closed isomer ***C***. The small hump around $E_p^{c} = -1.5$ V is assigned to residual oxygen in the reaction mixture.

Preparative Electrolysis

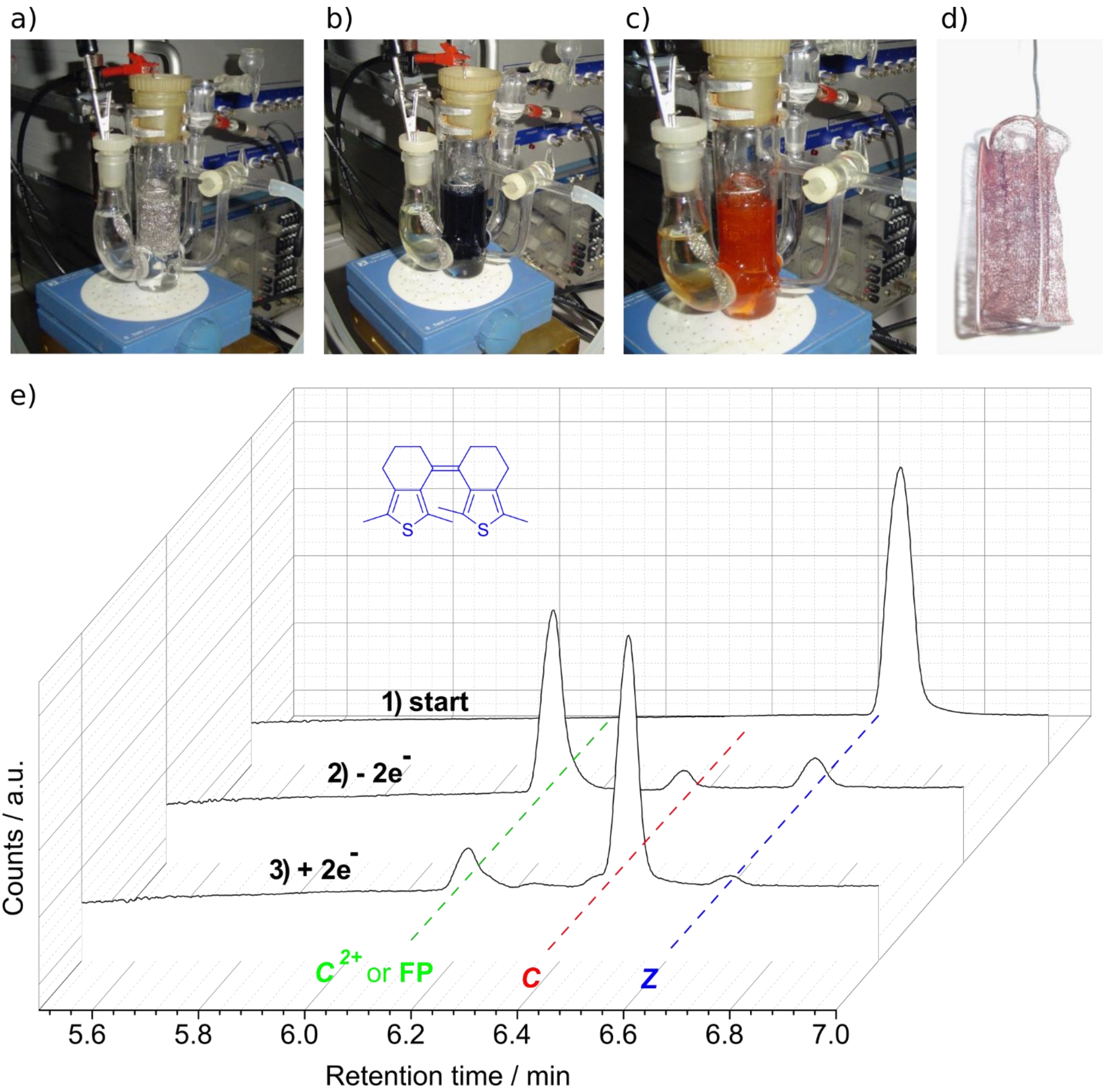

Figure S41: Preparative potentiostatic electrolysis of ***Z*-sDTE$_{66}$-Me**. Photographs of a) Initial solution b) after oxidation c) after re-reduction. The follow-up product partially stays in solution and d) partially deposits on the platin electrode as copper-colored film e) UPLC diode array traces of initial solution (***Z*-sDTE$_{66}$-Me** only), after oxidation (mainly **FP**) and final solution after filtration (mainly ***C*-sDTE$_{66}$-Me**, HR-MS (ESI$^+$) *m/z* = 329.1364 (calcd. 329.1398 for [$C_{20}H_{24}S_2$+H]$^+$)). All UPLC measurements with MeCN/H_2O (0.1% formic acid) as eluent.

<u>Procedure</u>: 7.932 mg of ***Z*-sDTE$_{66}$-Me** in 45 mL 0.1 M Bu_4NPF_6 in MeCN (trace 1), oxidation on a platin-net electrode in a double-H-cell at E = 1.0 V [SCE] (I_{start} = 20 mA, I_{end} = 1.25 mA; Q = 4950 mC ➔106 % for 2e$^-$/mol) (trace 2). Then re-reduction at E = -1.0 V [SCE] (I_{start} = -50 mA, I_{end} = -2.5 mA; Q = -4939 mC) (trace 3).

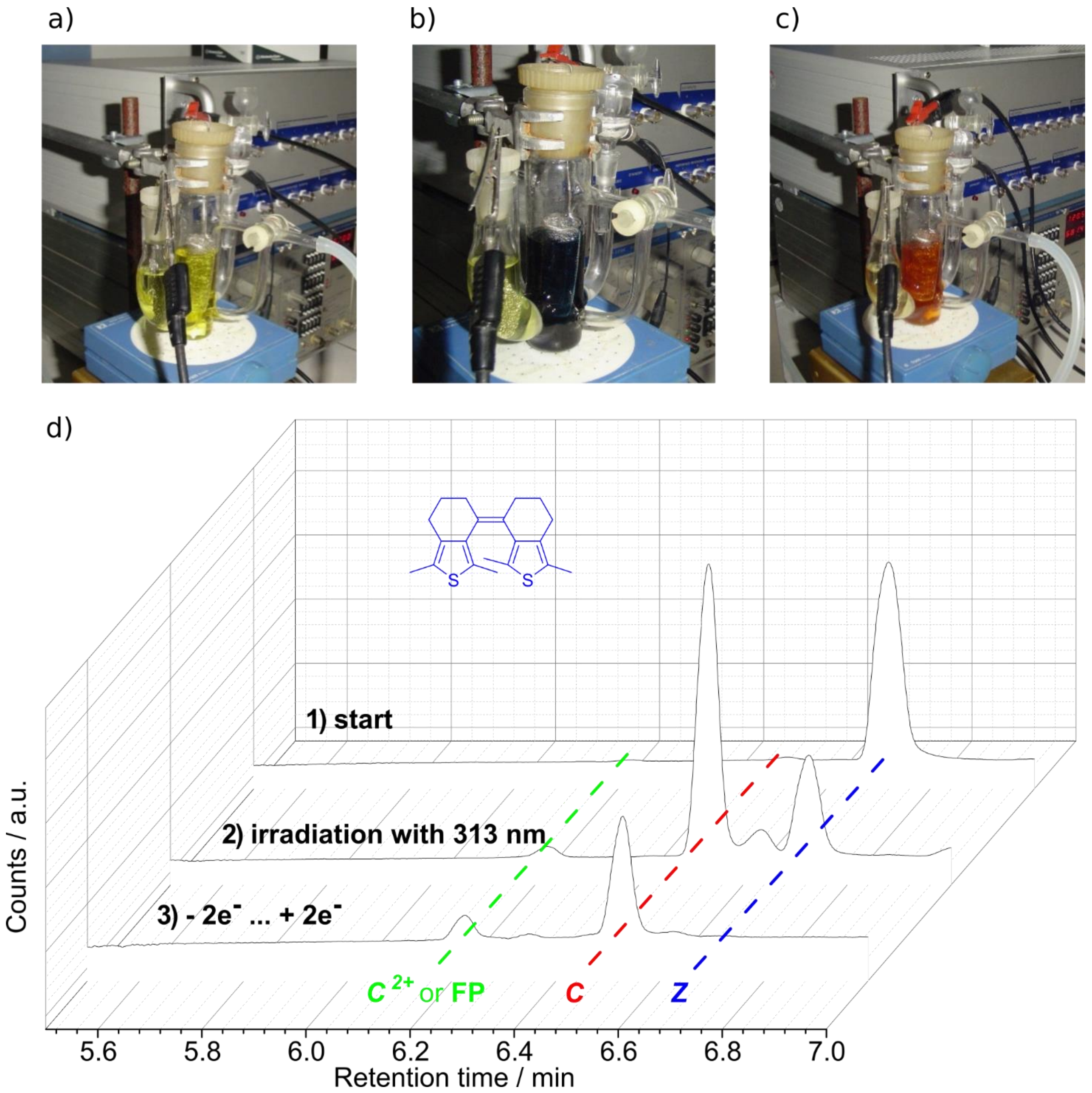

Figure S42: Preparative potentiostatic electrolysis of ***C*-sDTE$_{66}$-Me.** Photos of a) Initial solution of ***Z*-sDTE$_{66}$-Me** b) after irradiation with UV light c) after oxidation-reduction sequence. The follow-up product partially stays in solution and partially deposits on platin electrode as copper-colored film d) UPLC diode array trace of initial solution (***Z*-sDTE$_{66}$-Me** only), after irradiation (74% ***C*-sDTE$_{66}$-Me**) and final solution after filtration (mainly ***C*-sDTE$_{66}$-Me,** HR-MS (ESI$^+$) *m/z* = 329.1374 (calcd. 329.1398 for $[C_{20}H_{24}S_2+H]^+$)). All UPLC measurements with MeCN/H_2O (0.1% formic acid) as eluent.

<u>Procedure</u>: 5.719 mg of ***Z*-sDTE$_{66}$-Me** in 45 mL 0.1 M Bu_4NPF_6 in MeCN (trace 1), irradiated with 313 nm (TLC lamp) for 30 min in a double-H-cell (trace 2), then oxidation on a platin-net electrode in a double-H-cell at E = 0.8 V [SCE] (corresponding to ***C***$^{2+}$) (I_{start} = 21 mA, I_{end} = 1.0 mA; Q = 3120 mC ➔93 % for 2e$^-$/mol). Subsequent re-reduction at E = -0.9 V [SCE] (I_{start} = -40 mA, I_{end} = -2.0 mA; Q = -2800 mC) (trace 3).

a)

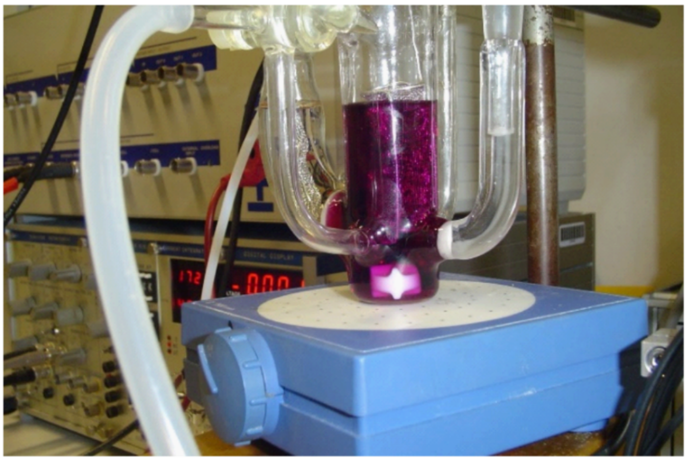

b)

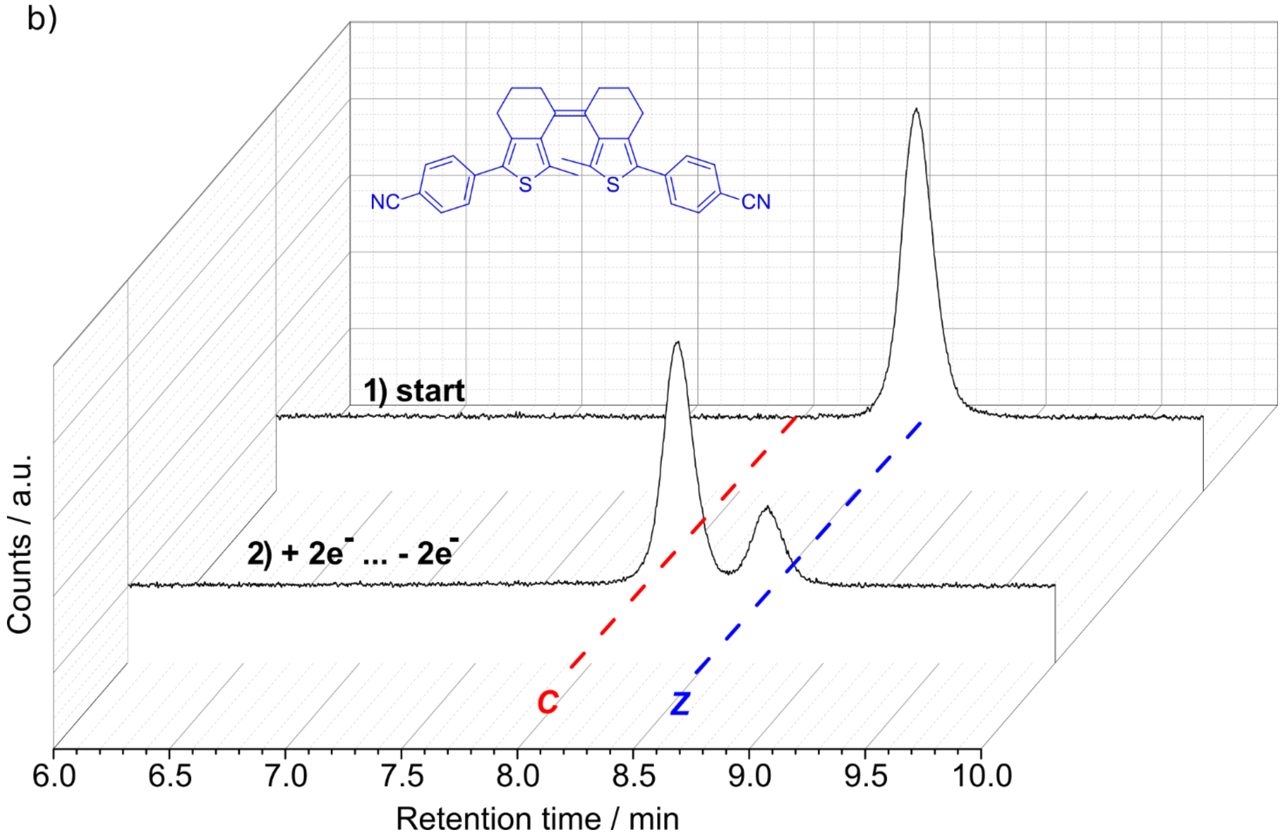

Figure S43: Preparative potentiostatic electrolysis of ***Z*-sDTE$_{66}$-PhCN**. a) Photo after reduction-oxidation sequence. b) UPLC diode array trace at isosbestic point of initial solution (***Z*-sDTE$_{66}$-PhCN** only) and final solution (mainly ***C*-sDTE$_{66}$-PhCN**, *C*:*Z* = 69:31). All UPLC measurements with MeCN/H_2O (0.1% formic acid) as eluent.

Procedure: 5.2 mg of ***Z*-sDTE$_{66}$-Me** in 45 mL 0.1 M Bu_4NPF_6 in MeCN (trace 1), filtrated through syringe filter before reduction on a platin-net electrode in a double-H-cell at E = -2.9 V vs. Fc/Fc$^+$ (Q = -3000 mC ➔150 % for 2e$^-$/mol). Then directly re-oxidation at E = -0.4 V vs. Fc/Fc$^+$ (Q = -1700 mC ➔85% for 2e$^-$/mol) (trace 2).

References

[1] L. N. Lucas, J. J. D. de Jong, J. H. van Esch, R. M. Kellogg, B. L. Feringa, *Eur. J. Org. Chem.* **2003**, *1*, 155–166.

[2] J.J.D. de Jong, L.N. Lucas, R. Hania, A. Pugzlys, R.M. Kellogg, B.L. Feringa, K. Duppen, J.H. van Esch, *Eur. J. Org. Chem.* **2003**, *2003*, 1887-1893.

[3] R. Grisorio, G.P. Suranna, P. Mastrorilli, M. Mazzeo, S. Colella, S. Carallo, G. Gigli, *Dalton Trans.* **2013**, *42*, 8939-8950.

[4] https://www.rhd-instruments.de/en/products/cells/tsc-1600-closed
